# Laboratory Evidence for Transovarial Transmission of *Babesia gibsoni* by *Haemaphysalis longicornis*

**DOI:** 10.1155/tbed/5585506

**Published:** 2026-09-15

**Authors:** Yaxin Zheng, Fangwei Chen, Jianyu Wang, Zhen Han, Sen Wang, Muxiao Li, Fen Du, Junlong Zhao, Lan He

**Affiliations:** ^1^ National Key Laboratory of Agricultural Microbiology, College of Veterinary Medicine, Huazhong Agricultural University, Wuhan 430070, Hubei Province, China, hzau.edu.cn; ^2^ Hubei International Scientific and Technological Cooperation Base of Veterinary Epidemiology, Huazhong Agricultural University, Wuhan 430070, China, hzau.edu.cn; ^3^ College of Animal Science and Technology, Tarim University, Alaer 843300, China, taru.edu.cn; ^4^ Engineering Laboratory of Tarim Animal Diseases Diagnosis and Control, Xinjiang Production and Construction Corps, Alaer 843300, China; ^5^ Center for Animal Disease Control and Prevention in Hubei Province, Wuhan 430070, Hubei Province, China; ^6^ Hubei Jiangxia Laboratory, Wuhan 430200, China

**Keywords:** *Babesia gibsoni*, *Haemaphysalis longicornis*, post-molt transmission, transovarial transmission, vector competence

## Abstract

*Babesia gibsoni* is a globally distributed tick‐borne apicomplexan parasite that threatens dog health, yet its transmission dynamics in *Haemaphysalis longicornis* remain incompletely defined. Under controlled laboratory conditions, a *Babesia*‐free parthenogenetic *H. longicornis* colony was used to evaluate transovarial transmission, transmission competence of the resulting F1 stages, retention of transmission competence after feeding on non‐susceptible rabbits, and post‐molt transmission after larval or nymphal acquisition feeding. Tick samples from the corresponding experimental lineages were screened by pooled internal transcribed spacer (ITS)‐targeted nested PCR at the egg, larval, nymphal, and adult stages. Transmission competence was evaluated by infesting splenectomized recipient dogs with the remaining ticks from the corresponding PCR‐screened lineages, followed by PCR and blood‐smear monitoring. Ticks from a PCR‐positive transovarial F1 lineage transmitted *B. gibsoni* to recipient dogs at the larval, nymphal, and adult stages. Within the transovarial F1 lineage, transmission competence was retained after one or two developmental‐stage feedings on non‐susceptible rabbits. In contrast, no evidence of post‐molt transmission to recipient dogs was observed after larval or nymphal acquisition feeding. Collectively, these findings provide laboratory evidence for transovarial transmission of *B. gibsoni* and transmission competence of the resulting F1 larval, nymphal, and adult stages in the tested parthenogenetic *H. longicornis* colony.

## 1. Introduction

*B. gibsoni* is a tick‐borne apicomplexan parasite that poses a serious threat to the health of dogs [1, 2]. Babesiosis in dogs caused by *B. gibsoni* has shown an increasing prevalence across East Asia in recent years [3–7]. Epidemiological studies indicate that, in addition to clinically affected dogs, apparently healthy dogs harbor subclinical infections, suggesting that vector‐mediated transmission is sustained in natural settings [6, 7]. Additionally, *B. gibsoni* has been detected in several tick species in East Asia, including *H. hystricis*, *H. bispinosa*, and the *Rhipicephalus sanguineus* sensu lato complex [8–11]. The Asian longhorned tick, *H. longicornis*, is widely distributed in *B. gibsoni*‐endemic regions and is considered the principal vector of this pathogen. This species is characterized by a broad host range and the capacity for parthenogenetic reproduction [6, 12–16]. Although numerous field surveys have reported frequent detection of *B. gibsoni* DNA in *H. longicornis*, it is important to distinguish between pathogen infection within the vector and true vector competence. The mere presence of pathogen DNA in ticks does not necessarily imply the ability to transmit the parasite to a vertebrate host during blood feeding [17–19].

Within the genus *Babesia*, transovarial transmission is generally considered an important mechanism for the long‐term maintenance of the parasite within tick populations and is often used to distinguish *Babesia* spp. from *Theileria* spp., which are typically maintained only through transstadial persistence [20, 21]. However, three related but distinct processes remain poorly understood for *B. gibsoni* in *H. longicornis*: transovarial transmission from infected adult female ticks to eggs and the resulting progeny; maintenance of transovarially acquired infection through larval, nymphal, and adult development; and horizontal acquisition during immature‐stage feeding, followed by post‐molt transmission. Moreover, the life cycle of *H. longicornis* frequently involves blood feeding on non‐susceptible hosts, such as lagomorphs and livestock [14, 16]. This raises an underexplored question: does feeding on a non‐susceptible host eliminate the transmission potential of a transovarial F1 lineage, or can transmission competence be retained at later developmental stages? Controlled laboratory studies are, therefore, needed to evaluate these processes separately. Non‐susceptible rabbits can be used to assess whether feeding on a non‐susceptible host affects subsequent transmission competence, whereas molecular detection combined with functional transmission assays in splenectomized dogs allows transmission to be assessed under highly susceptible recipient‐host conditions. Tracking *B. gibsoni* across successive developmental stages and experimental lineages is, therefore, necessary to distinguish PCR detection of parasite DNA in ticks from experimentally demonstrated transmission to recipient dogs and to determine how the acquisition route and tick developmental stage influence the vector competence of *H. longicornis*.

To address these knowledge gaps, this study experimentally evaluated transovarial transmission of *B. gibsoni* by *H. longicornis*, maintenance of transovarially acquired infection through the F1 larval, nymphal, and adult stages and the transmission competence of ticks at these stages, and retention of transmission competence after feeding on non‐susceptible rabbits. Post‐molt transmission after larval or nymphal acquisition feeding was also assessed. Tick lineages were screened by pooled nested PCR, and transmission was evaluated using splenectomized recipient dogs under controlled laboratory conditions.

## 2. Materials and Methods

### 2.1. Parasites, Ticks, and Animals

The *B. gibsoni* WH II strain, a clinical isolate obtained in 2023 from a pet dog presenting with severe clinical babesiosis, was used for all transmission experiments. This strain is continuously maintained and cryopreserved in liquid nitrogen at the Parasitology Laboratory of Huazhong Agricultural University (Wuhan, China). Prior to experimental use, the cryopreserved parasites were reactivated in splenectomized Beagle donor dogs by administering half of the inoculum intravenously (IV) and the other half subcutaneously (SC) to establish parasitemia.

A *Babesia*‐free parthenogenetic colony of *H. longicornis* ticks (Cangxi strain, Sichuan, China) was kindly provided by Qinghai University. The tick colony was routinely maintained by feeding on Japanese White rabbits (*Oryctolagus cuniculus*). Off‐host tick stages were housed in an incubator at 26°C with a 70%–90% relative humidity.

Healthy experimental Beagle dogs (6–8 months old) were purchased from Wuhan Origin of Life Technology Co., Ltd. (Wuhan, China). Prior to the study, all dogs were confirmed negative for *B. gibsoni* via microscopic examination of Giemsa‐stained peripheral blood smears and internal transcribed spacer (ITS)‐targeted PCR. Splenectomies were performed at the Veterinary Teaching Hospital 1–2 weeks before the initial *B. gibsoni* infection or tick infestation.

To minimize animal suffering, all surgical procedures were performed under strict aseptic conditions. Before splenectomy, the dogs were fasted for 12 h. As a preanesthetic medication, atropine 0.04 mg/kg was administered SC to reduce salivary secretions and prevent vagal reflex‐induced bradycardia. General anesthesia was then achieved with an intramuscular injection of Zoletil (5 mg/kg). Postoperative care and daily intensive monitoring were conducted to ensure animal welfare, alleviate any discomfort, and facilitate recovery.

### 2.2. DNA Extraction and Purification

Genomic DNA was extracted from the peripheral blood of dogs, as well as tick eggs, larvae, nymphs, and adults, using the TIANamp Genomic DNA Kit (Tiangen Biotech, Beijing, China). While blood samples were processed strictly according to the manufacturer’s standard protocol, tick samples across all developmental stages were subjected to an extended proteinase K digestion for 3–6 h prior to column‐based purification. The purified genomic DNA was eluted in 50 μL of TB buffer and stored for subsequent PCR amplification.

### 2.3. ITS‐Based PCR Detection

All samples were screened for the presence of *B. gibsoni* using a two‐round nested PCR targeting the ITS region, as previously described [4]. Both PCR rounds were performed in a 25 μL reaction volume containing 20 μL of Tsingke Golden Mix (Green) (Tsingke Biotechnology, Beijing, China) and 1 μL each of the forward and reverse primers. The primary reaction contained 3 μL of the genomic DNA template, whereas the second‐round reaction contained 1 μL of the first‐round amplicon and 2 μL of double‐distilled water. The primer sequences (5′–3′) were BgITS1‐F (GGTGGTGCATGGCCG) and BgITS1‐R (TGCGCTTCAATCCC) for the primary amplification and BgITS2‐F (GAGAAGTCGTAACAAGGTTTCCG) and BgITS2‐R (GCTTCACTCGCCGTTACTAGG) for the second round. Before and after the first‐ and second‐round PCR procedures, the relevant working areas and materials were exposed to ultraviolet irradiation for 30 min. Template addition for the second round was performed in a laminar‐flow clean bench, with tubes opened one at a time. For each DNA extraction batch, *B. gibsoni*‐infected erythrocyte material and *Babesia*‐free egg or tick material were processed in parallel as the positive extraction control and batch‐matched mock negative extraction control, respectively. The *Babesia*‐free egg or tick material was carried through homogenization, DNA extraction, and both rounds of PCR using the same amplification reagents and experimental setup as the study samples, thereby serving as an end‐to‐end negative process control. PCR results were accepted only when the corresponding batch‐matched mock negative extraction control remained negative. Genomic DNA extracted from *B. gibsoni*‐infected erythrocytes derived from infected dog blood or in vitro culture served as the positive PCR control. Because the batch‐matched mock negative extraction control contained a *Babesia*‐free biological matrix and its extract was used as the PCR template, it was not equivalent to a reagent‐only extraction blank or a PCR no‐template control. Neither a separate reagent‐only extraction blank nor a separate no‐template control was included. Thermal cycling was conducted on a Bio‐Rad thermal cycler with the following conditions: initial denaturation at 98°C for 2 min, followed by 30 cycles of 98°C for 10 s, annealing for 30 s (51.2°C for the primary PCR and 60°C for the second‐round PCR), and 72°C for 30 s, concluding with a final extension at 72°C for 5 min. The expected second‐round amplicon was 914 bp, and the products were resolved by electrophoresis on a 1.5% agarose gel stained with ethidium bromide and visualized using a Gel Doc 2000 imaging system (Bio‐Rad).

### 2.4. Sequence Verification of Representative PCR Products

Twenty‐one representative positive 914 bp second‐round ITS amplicons were selected for single‐direction Sanger sequencing using the BgITS2‐F primer (Sangon Biotech, Shanghai, China). These included two amplicons from each of the PCR‐positive Eggs‐1, Larvae‐1, Nymphs‐1, Adults‐1, Larvae‐2, Nymphs‐2, and Adults‐2 groups; one amplicon from the first PCR‐positive blood sample from each donor dog; and one amplicon from the first PCR‐positive blood sample from each PCR‐positive recipient dog (T01–T05). The resulting chromatograms were opened and visually inspected in SnapGene software, and the obtained sequences were compared by BLAST with the verified ITS reference sequence of the cryopreserved and reactivated *B. gibsoni* WH II strain used as the experimental inoculum. A BLAST‐based comparison showed 99.8%–100% identity with the WH II ITS reference sequence, verifying the representative amplicons as *B. gibsoni* ITS sequences.

### 2.5. Clinical and Molecular Monitoring

A standardized clinical and molecular monitoring protocol was strictly implemented for all in vivo transmission experiments in dogs. Following tick infestation, rectal temperatures and the status of tick attachment within the ear bags were recorded daily starting on day 1 post‐infestation (1 dpi). For molecular detection, peripheral blood samples were collected every other day beginning at 3 dpi. Upon the initial positive PCR result, an additional blood sample was collected and tested the following day for confirmation. Once PCR positivity was confirmed, subsequent monitoring was performed by daily examination of Giemsa‐stained thin blood smears rather than by serial PCR. For dogs that remained PCR‐negative, Giemsa‐stained thin blood smears were examined concurrently with each scheduled PCR test, and both PCR and blood smear monitoring continued through 31 dpi. Ten microscopic fields were examined for each smear, corresponding to approximately 6000 erythrocytes. A smear was considered positive when at least one morphologically identifiable intraerythrocytic parasite was observed. Parasitemia was calculated as the percentage of parasitized erythrocytes among the total erythrocytes examined.

### 2.6. Transmission of *B. gibsoni* From Dogs to Ticks

To establish parasitemia in donor dogs, 1 × 10^8^ *B. gibsoni* WH II‐infected erythrocytes suspended in physiological saline were administered, with half of the inoculum delivered IV and the other half SC. Parasitemia was monitored daily by microscopic examination of Giemsa‐stained thin blood smears.

To facilitate tick attachment and feeding, the hair around the base of each dog’s ears was shaved, and breathable cotton ear bags were secured using a custom gelatin‐zinc oxide‐water‐glycerol adhesive mixed at a ratio of 3:3:3:2 (w/w/w/w). Unfed ticks were introduced into the ear bags, and tick attachment and engorgement were monitored daily.

Separate infected donor dogs were used for the adult female tick acquisition experiment and the larval and nymphal acquisition experiments. For the transovarial line, 80 *Babesia*‐free adult female ticks were placed on Donor dog 1 on day 9 post‐inoculation, when parasitemia was 1.0%. Parasitemia values on days 9–16 post‐inoculation were 1.0%, 2.0%, <1.0%, 10.0%, 15.0%, 11.2%, 10.0%, and 1.0%, respectively. Thirty‐four engorged adult females were recovered after natural detachment during days 12–16 post‐inoculation. Donor dog 1 remained smear‐positive when the final engorged adult females were recovered, with a parasitemia of 1.0% on day 16 post‐inoculation.

For the larval and nymphal acquisition experiments, approximately 7000–8000 *Babesia*‐free larvae and 1000 *Babesia*‐free nymphs were placed simultaneously on Donor dog 2 on day 12 post‐inoculation, when parasitemia was 1.5%. Parasitemia values on days 12–19 post‐inoculation were 1.5%, 1.5%, 6.4%, 5.9%, 5.9%, 7.3%, 14.6%, and 10.2%, respectively. A total of 2664 engorged larvae and 852 engorged nymphs were recovered after natural detachment during days 16–19 post‐inoculation. Additional details are provided in Supporting Information 1: Table S1A.

Naturally detached, fully engorged ticks were carefully collected. Fully engorged larvae and nymphs were incubated at 26°C and 70%–90% relative humidity to allow molting to the subsequent developmental stage, whereas fully engorged adult females were maintained under the same conditions to allow oviposition. Because ticks within the same infestation did not engorge synchronously, the engorged tick collection period was recorded as the interval from the first to the final recovery of fully engorged ticks. The overall infestation period was calculated from the initial tick placement to the final recovery of fully engorged ticks and represents the group‐level experimental period rather than the feeding duration of an individual tick. The resulting post‐molt stages, egg batches, and hatched larvae were used for pooled PCR screening or subsequent transmission experiments, as described below.

### 2.7. Transovarial Transmission Experiments

#### 2.7.1. Experimental Line 1: Transmission Competence Across All Tick Developmental Stages

Unfed adult female ticks were allowed to feed on *B. gibsoni*‐infected Donor dog 1 until full engorgement. Following oviposition and hatching, the first filial (F1) generation was obtained. PCR screening was destructive and was therefore performed on subsets of ticks from each experimental lineage. Where transmission was assessed, the remaining ticks from the same lineage were used to infest recipient dogs. Thus, the recipient dogs were exposed to ticks from the same PCR‐screened lineage. To assess transovarial transmission, subsets of F1 eggs (100 eggs per pool) and unfed larvae (20 larvae per pool) were homogenized by grinding in liquid nitrogen, and their genomic DNA was extracted and screened by PCR, as described above.

The remaining F1 larvae were used to infest naive splenectomized dogs. Upon engorgement and molting to nymphs, a subset of F1 nymphs was screened by PCR (10 nymphs per pool), and the remaining nymphs were used to infest naive splenectomized dogs. After molting to adults, a subset of F1 adults (five adults per pool) was tested by PCR, and the remaining adults were fed on naive splenectomized dogs to assess transmission competence at each developmental stage.

#### 2.7.2. Experimental Line 2: Transmission Competence Following Feeding on a Non‐Susceptible Host (Rabbits)

To evaluate the impact of a non‐susceptible host on parasite persistence, F1 larvae derived from infected adult ticks (as generated in Line 1) were fed on 12‐week‐old Japanese white rabbits. The rabbits were prepared by shaving the dorsal area, and cloth feeding bags were attached using the aforementioned gelatin‐based adhesive. After engorgement and molting to F1 nymphs, a subset was screened by PCR, and the remaining nymphs were used to infest naive splenectomized dogs to evaluate the transmission competence.

In a parallel assay to test consecutive non‐susceptible feedings, F1 larvae were first fed on rabbits, and the resulting F1 nymphs were subsequently fed on rabbits again. After molting to F1 adults, a subset was screened by PCR, and the remaining adults were used to infest naive splenectomized dogs to assess whether transmission competence was retained after continuous development exclusively on non‐susceptible hosts.

### 2.8. Larval and Nymphal Acquisition Followed by Post‐Molt Transmission Assessment

To assess post‐molt transmission after larval or nymphal acquisition feeding, two experimental lines were established. PCR screening was performed after the acquisition‐fed larvae and nymphs had molted to the subsequent developmental stage.

In the larval acquisition line, *Babesia*‐free unfed larvae were allowed to feed on *B. gibsoni*‐infected Donor dog 2. After engorgement and molting to nymphs, a subset of the resulting nymphs was screened by PCR, whereas the remaining nymphs from the same experimental line were allowed to feed on a *B. gibsoni*‐negative splenectomized recipient dog. After these nymphs molted to adults, a subset of the resulting adults was screened by PCR, whereas the remaining adults were allowed to feed on another *B. gibsoni*‐negative splenectomized recipient dog. Following engorgement and oviposition, the resulting eggs and hatched larvae were screened by PCR, and the remaining larvae from the same lineage were allowed to feed on a *B. gibsoni*‐negative splenectomized recipient dog to assess potential next‐generation transmission.

In the nymphal acquisition line, *Babesia*‐free unfed nymphs were allowed to feed on *B. gibsoni*‐infected Donor dog 2. After engorgement and molting to adults, a subset of the resulting adults was screened by PCR, whereas the remaining adults from the same experimental line were allowed to feed on a *B. gibsoni*‐negative splenectomized recipient dog. Following engorgement and oviposition, the resulting eggs and hatched larvae were screened by PCR, and the remaining larvae from the same lineage were allowed to feed on a *B. gibsoni*‐negative splenectomized recipient dog to assess potential next‐generation transmission.

## 3. Results

### 3.1. Transovarial Transmission and Transmission Competence of the F1 Lineage at the Larval, Nymphal, and Adult Stages

*Babesia*‐free F0 adult female *H. longicornis* ticks were placed on Donor dog 1 on day 9 post‐inoculation, when parasitemia was 1.0%. Eighty adult females were applied, and 34 engorged females were recovered after natural detachment during days 12–16 post‐inoculation and maintained for oviposition. Pooled PCR detected *B. gibsoni* DNA in 22/34 Eggs‐1 pools and 34/34 unfed Larvae‐1 pools. Detailed donor dog parasitemia and tick acquisition data are provided in Supporting Information 1: Table S1A.

The remaining Larvae‐1 ticks from the same experimental lineage were placed on the splenectomized recipient dog T01. T01 was first PCR‐positive at 5 dpi and first smear‐positive at 10 dpi, with a parasitemia of 0.01% at the first smear‐positive time point. A total of 1398 engorged larvae were recovered and allowed to molt into Nymphs‐1.

Before recipient‐dog infestation, pooled PCR detected *B. gibsoni* DNA in 10/10 unfed Nymphs‐1 pools. The remaining Nymphs‐1 ticks from the same experimental lineage were placed on the splenectomized recipient dog, T02. T02 was first PCR‐positive at 7 dpi and first smear‐positive at 10 dpi, with a parasitemia of 0.12% at the first smear‐positive time point. A total of 445 engorged nymphs were recovered and allowed to molt into Adults‐1.

Before recipient‐dog infestation, pooled PCR detected *B. gibsoni* DNA in 3/11 unfed Adults‐1 pools. The remaining Adults‐1 females from the same experimental lineage were placed on the splenectomized recipient dog, T03. T03 was first PCR‐positive at 11 dpi and first smear‐positive at 15 dpi, with a parasitemia of 0.10% at the first smear‐positive time point. A total of 41 engorged adult females were recovered.

**Figure 1:**
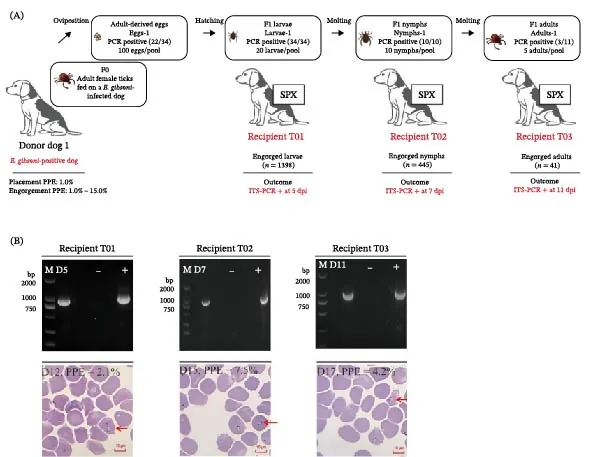
Laboratory evidence for transovarial transmission of *B. gibsoni* and transmission competence of the F1 *H. longicornis* lineage at the larval, nymphal, and adult stages. (A) *Babesia*‐free F0 adult female ticks were allowed to feed on *B. gibsoni* WH II‐infected Donor dog 1 until engorgement and were subsequently maintained for oviposition, generating the Eggs‐1 and Larvae‐1 groups. Subsets of Eggs‐1 and Larvae‐1 were screened by pooled PCR, whereas the remaining Larvae‐1 ticks from the same lineage were allowed to feed on the splenectomized recipient dog, T01. After molting, subsets of the resulting Nymphs‐1 and Adults‐1 groups were screened by pooled PCR, whereas the remaining ticks from the same lineage were allowed to feed on splenectomized recipient dogs T02 and T03, respectively. (B) ITS‐targeted nested PCR results from the first PCR‐positive blood samples of recipient dogs T01, T02, and T03, together with representative Giemsa‐stained thin blood smear images showing intraerythrocytic parasites (arrows). Because parasitemia was very low at the first smear‐positive time points, representative smear images were selected from later smear‐positive samples in which parasites could be more clearly observed: T01 at D12 (PPE = 2.1%), T02 at D15 (PPE = 7.5%), and T03 at D17 (PPE = 4.2%). The actual sampling day and PPE are indicated on each image. M, DNA marker; (−), batch‐matched mock negative extraction control; (+), positive PCR control; D, days post‐infestation, with the day of tick placement defined as D0; PPE, percentage of parasitized erythrocytes; SPX, splenectomized. Scale bar: 10 μm. Transmission from engorged F1 Adults‐1 females to F2 eggs or larvae was not evaluated.

Pool‐level PCR results and recipient‐dog transmission outcomes are summarized in Table 1. Collectively, these findings provide laboratory evidence that ticks from a PCR‐positive transovarial F1 lineage transmitted *B. gibsoni* to splenectomized recipient dogs at the larval, nymphal, and adult stages (Figure 1 and Table 1). Raw gel images and pool‐level PCR outcomes for the corresponding tick groups and recipient dogs are provided in Supporting Information 2: Figure S1. Tick‐group definitions and recipient dog details are summarized in Supporting Information 1: Table S1B,C.

**Table 1:** Pool‐level PCR detection of *B. gibsoni* in transovarial progeny and transmission evidence at the larval, nymphal, and adult stages.

Tick stage	Pooled sample size	PCR‐positive pools/total pools (%)	Transmission to recipient dog (s)	Evidence (recipient)
Eggs‐1	(100 eggs/pool)	22/34 = 64.7	Not assessed	NA
Larvae‐1	(20 larvae/pool)	34/34 = 100	Yes (dog ID: T01)	PCR‐positive at 5 dpi; smear‐positive from 10 dpi
Nymphs‐1	(10 nymphs/pool)	10/10 = 100	Yes (dog ID: T02)	PCR‐positive at 7 dpi; smear‐positive from 10 dpi
Adults‐1	(5 adults/pool)	3/11 = 27.3	Yes (dog ID: T03)	PCR‐positive at 11 dpi; smear‐positive from 15 dpi

*Note*: PCR positivity was determined by ITS‐targeted nested PCR and is reported as PCR‐positive pools/total pools tested (%). Transmission to recipient dogs: yes indicates that transmission was detected, as the recipient dog became PCR‐positive during the observation period; NA indicates that transmission was not directly assessed for that tick group. Eggs, larvae, nymphs, and adults were tested in pools of 100 eggs, 20 larvae, 10 nymphs, and 5 adults, respectively. Pool‐level PCR positivity does not represent individual tick infection prevalence, and percentages should not be directly compared across developmental stages because pool sizes differed. PCR screening was performed on subsets of ticks from each experimental lineage, whereas the remaining ticks from the same lineage were used for recipient‐dog infestation; therefore, the PCR‐tested pools and the ticks placed on recipient dogs did not comprise the same individual ticks.

Abbreviation: dpi, days post‐infestation.

### 3.2. Transmission Competence of the Transovarial F1 Lineage After Feeding on Non‐Susceptible Rabbits

In the first experimental line, pooled PCR detected *B. gibsoni* DNA in 5/5 unfed Larvae‐2 pools derived from the Eggs‐1 group. The remaining Larvae‐2 ticks from the same transovarial lineage were allowed to feed to engorgement on non‐susceptible rabbits. Following engorgement and molting, pooled PCR detected *B. gibsoni* DNA in 1/5 unfed Nymphs‐2 pools. The remaining Nymphs‐2 ticks from the same experimental lineage were placed on the splenectomized recipient dog, T04. T04 was first PCR‐positive at 7 dpi and first smear‐positive at 11 dpi, with a parasitemia of 0.40% at the first smear‐positive time point. A total of 643 engorged nymphs were recovered from T04.

In a parallel experimental branch, the remaining Nymphs‐2 ticks were allowed to feed to engorgement on rabbits and subsequently molt into Adults‐2. Before recipient‐dog infestation, pooled PCR detected *B. gibsoni* DNA in 3/10 unfed Adults‐2 pools. The remaining Adults‐2 females from the same experimental lineage were placed on the splenectomized recipient dog, T05. T05 was first PCR‐positive at 9 dpi and first smear‐positive at 11 dpi, with a parasitemia of 0.11% at the first smear‐positive time point. A total of 60 engorged adult females were recovered from T05.

**Figure 2:**
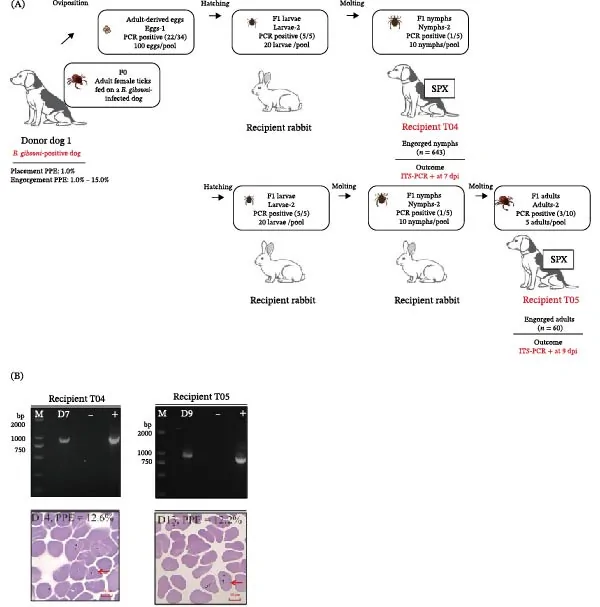
Transmission competence of the transovarial F1 *H. longicornis* lineage after feeding on non‐susceptible rabbits. (A) Experimental design used to evaluate whether ticks from the transovarial F1 lineage retained transmission competence after feeding on non‐susceptible rabbits. Larvae‐2 ticks derived from Eggs‐1 were allowed to feed on rabbits and molt into Nymphs‐2. Subsets of the resulting Nymphs‐2 were screened by pooled PCR, whereas the remaining nymphs from the same lineage were allowed to feed on the splenectomized recipient dog, T04. In the second experimental line, Nymphs‐2 were allowed to feed on rabbits and molt into Adults‐2. Subsets of Adults‐2 were screened by pooled PCR, whereas the remaining adults from the same lineage were allowed to feed on the splenectomized recipient dog, T05. (B) ITS‐targeted nested PCR results from the first PCR‐positive blood samples of recipient dogs T04 and T05, together with representative Giemsa‐stained thin blood smear images showing intraerythrocytic parasites (arrows). Because parasitemia was low at the first smear‐positive time points, representative smear images were selected from later smear‐positive samples in which parasites could be more clearly observed: T04 at D14 (PPE = 12.6%) and T05 at D13 (PPE = 12.2%). The actual sampling day and PPE are indicated on each image. M, DNA marker; (−), batch‐matched mock negative extraction control; (+), positive PCR control; D, days post‐infestation, with the day of tick placement defined as D0; PPE, percentage of parasitized erythrocytes; SPX, splenectomized. Scale bar: 10 μm.

Pool‐level PCR results and recipient‐dog transmission outcomes are summarized in Table 2. Collectively, under the tested laboratory conditions, ticks from the transovarial F1 lineage transmitted *B. gibsoni* to splenectomized recipient dogs after feeding on non‐susceptible rabbits during the larval stage or during both the larval and nymphal stages. These findings indicate that rabbit feeding did not eliminate transmission competence in the corresponding experimental lineages (Figure 2 and Table 2). Raw gel images and pool‐level PCR outcomes for the corresponding tick groups and recipient dogs are provided in Supporting Information 2: Figure S1. Recipient‐dog infestation, tick recovery, and monitoring details are summarized in Supporting Information 1: Table S1C.

**Table 2:** Pool‐level PCR detection of *B. gibsoni* in transovarial progeny after feeding on non‐susceptible rabbits and transmission evidence at the nymphal and adult stages.

Tick stage	Pooled sample size	PCR‐positive pools/total pools (%)	Transmission to recipient dog (s)	Evidence (recipient)
Larvae‐2	(20 larvae/pool)	5/5 = 100	NA (fed on rabbit)	NA
Nymphs‐2	(10 nymphs/pool)	1/5 = 20	Yes (dog ID: T04)	PCR‐positive at 7 dpi; smear‐positive from 11 dpi
Adults‐2	(5 adults/pool)	3/10 = 30	Yes (dog ID: T05)	PCR‐positive at 9 dpi; smear‐positive from 11 dpi

*Note*: PCR positivity was determined by ITS‐targeted nested PCR and is reported as PCR‐positive pools/total pools tested (%). Transmission to recipient dogs: yes indicates that transmission was detected, as the recipient dog became PCR‐positive during the observation period; NA indicates that the corresponding tick group was not directly assessed by recipient‐dog infestation, including rabbit‐fed stages used to generate the subsequent developmental stage. Larvae, nymphs, and adults were tested in pools of 20, 10, and 5 ticks, respectively. Pool‐level PCR positivity does not represent individual tick infection prevalence, and percentages should not be directly compared across developmental stages because pool sizes differed. PCR screening was performed on subsets of ticks from each experimental lineage, whereas the remaining ticks from the same lineage were used for recipient‐dog infestation; therefore, the PCR‐tested pools and the ticks placed on recipient dogs did not comprise the same individual ticks.

Abbreviation: dpi, days post‐infestation.

### 3.3. No Evidence of Post‐Molt Transmission After Larval or Nymphal Acquisition Feeding

To assess whether larvae or nymphs that fed on an infected donor dog subsequently transmitted *B. gibsoni* after molting, two experimental lines were evaluated. Donor dog 2 was smear‐positive at tick placement and throughout the engorged‐tick collection period, confirming that the larvae and nymphs fed under parasitemic conditions. In the larval acquisition line, *Babesia*‐free larvae were allowed to feed to engorgement on *B. gibsoni*‐infected Donor dog 2 and subsequently molt into Nymphs‐3. Before recipient‐dog infestation, pooled PCR detected no *B. gibsoni* DNA in any of the 10 unfed Nymphs‐3 pools. The remaining Nymphs‐3 ticks from the same experimental lineage were placed on the splenectomized recipient dog, T06. T06 remained PCR‐negative at every tested time point from 3 through 31 dpi and smear‐negative at all corresponding monitoring time points through 31 dpi. A total of 535 engorged nymphs were recovered from T06 and allowed to molt into Adults‐3. Before recipient‐dog infestation, pooled PCR detected no *B. gibsoni* DNA in any of the 10 unfed Adults‐3 pools. The remaining Adults‐3 females from the same experimental lineage were placed on the splenectomized recipient dog, T07. T07 remained PCR‐negative at every tested time point from 3 through 31 dpi and smear‐negative at all corresponding monitoring time points through 31 dpi. A total of 80 engorged adult females were recovered from T07 and maintained for oviposition. Pooled PCR detected no *B. gibsoni* DNA in any of the 71 Eggs‐2 pools. After hatching, pooled PCR also detected no *B. gibsoni* DNA in any of the 10 unfed Larvae‐3 pools. The remaining Larvae‐3 ticks from the same experimental lineage were placed on the splenectomized recipient dog, T08. T08 remained PCR‐negative at every tested time point from 3 through 31 dpi and smear‐negative at all corresponding monitoring time points through 31 dpi. A total of 3468 engorged larvae were recovered from T08.

In the nymphal acquisition line, *Babesia*‐free nymphs were allowed to feed to engorgement on *B. gibsoni*‐infected Donor dog 2 and subsequently molt into Adults‐4. Before recipient‐dog infestation, pooled PCR detected no *B. gibsoni* DNA in any of the 10 unfed Adults‐4 pools. The remaining Adults‐4 females from the same experimental lineage were placed on the splenectomized recipient dog, T09. T09 remained PCR‐negative at every tested time point from 3 through 31 dpi and smear‐negative at all corresponding monitoring time points through 31 dpi. A total of 95 engorged adult females were recovered from T09 and maintained for oviposition. Pooled PCR detected no *B. gibsoni* DNA in any of the 54 Eggs‐3 pools. After hatching, pooled PCR also detected no *B. gibsoni* DNA in any of the 10 unfed Larvae‐4 pools. The remaining Larvae‐4 ticks from the same experimental lineage were placed on the splenectomized recipient dog, T10. T10 remained PCR‐negative at every tested time point from 3 through 31 dpi and smear‐negative at all corresponding monitoring time points through 31 dpi. A total of 793 engorged larvae were recovered from T10.

**Figure 3:**
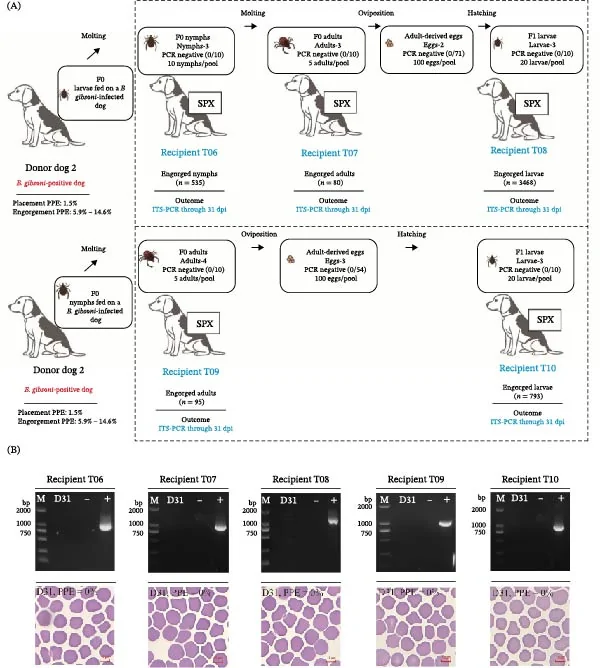
Assessment of post‐molt transmission following larval or nymphal acquisition feeding. (A) *Babesia*‐free larvae or nymphs were allowed to feed on a *B. gibsoni*‐infected donor dog and subsequently molt into nymphs (Nymphs‐3) or adults (Adults‐4), respectively. In the larval acquisition line, Nymphs‐3, adults derived from Nymphs‐3 (Adults‐3), and progeny larvae hatched from eggs laid by Adults‐3 (Eggs‐2→Larvae‐3) were used to infest splenectomized recipient dogs T06–T08, respectively. In the nymphal acquisition line, Adults‐4 and progeny larvae hatched from eggs laid by Adults‐4 (Eggs‐3→Larvae‐4) were used to infest splenectomized recipient dogs T09 and T10, respectively. Pooled tick ITS‐PCR results are presented as PCR‐positive pools/total pools tested, and all recipient dogs remained ITS‐PCR‐negative throughout the monitoring period. PCR screening of the acquisition‐fed larvae and nymphs was performed only after they had molted to the subsequent developmental stage. (B) Representative blood ITS‐PCR results from recipient dogs at the indicated days post‐infestation, together with representative Giemsa‐stained thin blood smear images showing no detectable intraerythrocytic parasites. M, DNA marker; (−), batch‐matched mock negative extraction control; (+), positive PCR control; dpi, days post‐infestation, with the day of tick placement defined as 0 dpi; SPX, splenectomized. Scale bar: 10 μm.

Pool‐level PCR results and recipient dog outcomes for both experimental lines are summarized in Table 3. Collectively, under the laboratory conditions and experimental design used in this study, no evidence of post‐molt transmission was observed after larval or nymphal acquisition feeding. Transmission was likewise not detected from the subsequent developmental stages or from the resulting F1 larvae (Figure 3 and Table 3). Raw gel images and pool‐level PCR outcomes for the corresponding tick groups and recipient dogs are provided in Supporting Information 2: Figure S2. Recipient dog infestation, tick recovery, and monitoring details are summarized in Supporting Information 1: Table S1C.

**Table 3:** Pool‐level PCR detection of *B. gibsoni* following larval or nymphal acquisition feeding and transmission evidence at post‐molt stages and in F1 larvae.

Tick stage	Pooled sample size	PCR‐positive pools/total pools (%)	Transmission to recipient dog (s)	Evidence (recipient)
Nymphs‐3	(10 nymphs/pool)	0/10 = 0	No (dog ID: T06)	PCR‐negative through 31 dpi; smear‐negative at all corresponding monitoring time points through 31 dpi
Adults‐3	(5 adults/pool)	0/10 = 0	No (dog ID: T07)	PCR‐negative through 31 dpi; smear‐negative at all corresponding monitoring time points through 31 dpi
Eggs‐2	(100 eggs/pool)	0/71 = 0	NA	NA
Larvae‐3	(20 larvae/pool)	0/10 = 0	No (dog ID: T08)	PCR‐negative through 31 dpi; smear‐negative at all corresponding monitoring time points through 31 dpi
Adults‐4	(5 adults/pool)	0/10 = 0	No (dog ID: T09)	PCR‐negative through 31 dpi; smear‐negative at all corresponding monitoring time points through 31 dpi
Eggs‐3	(100 eggs/pool)	0/54 = 0	NA	NA
Larvae‐4	(20 larvae/pool)	0/10 = 0	No (dog ID: T10)	PCR‐negative through 31 dpi; smear‐negative at all corresponding monitoring time points through 31 dpi

*Note*: PCR positivity was determined by ITS‐targeted nested PCR and is reported as PCR‐positive pools/total pools tested (%). Transmission to recipient dogs: no indicates that the recipient dog remained PCR‐negative throughout the monitoring period; NA indicates that the corresponding tick group was not directly assessed by recipient‐dog infestation, including egg stages. Eggs, larvae, nymphs, and adults were tested in pools of 100 eggs, 20 larvae, 10 nymphs, and 5 adults, respectively. PCR screening of acquisition‐fed larvae and nymphs was performed after they had molted to the subsequent developmental stage. Pool‐level PCR positivity does not represent individual tick infection prevalence, and percentages should not be directly compared across developmental stages because pool sizes differed. PCR screening was performed on subsets of ticks from each experimental lineage, whereas the remaining ticks from the same lineage were used for recipient‐dog infestation; therefore, the PCR‐tested pools and the ticks placed on recipient dogs did not comprise the same individual ticks.

Abbreviation: dpi, days post‐infestation.

## 4. Discussion

This study provides laboratory evidence for transovarial transmission of *B. gibsoni* by the tested parthenogenetic *H. longicornis* colony. Ticks from a PCR‐positive transovarial F1 lineage transmitted *B. gibsoni* to splenectomized recipient dogs at the larval, nymphal, and adult stages. These findings support the maintenance of transovarially acquired infection through F1 development and transmission competence at multiple developmental stages. PCR screening was performed on pooled subsets of ticks, whereas the remaining ticks from the same experimental lineages were used for recipient dog infestation. In addition, one recipient dog was used for each tick stage or experimental line; therefore, the findings provide qualitative proof of principle but do not quantify individual tick infection prevalence, transmission efficiency, or stage‐specific transmission probability.

Transovarial transmission by *Babesia* spp. is generally considered to require parasite developmental stages to traverse tick tissues and reach the ovaries and developing oocytes. Migration through the hemolymph and invasion of the ovaries have been described as components of the tick stage development of *Babesia* spp. [22]. However, the present study did not examine the tick midgut, hemolymph, ovaries, salivary glands, or specific parasite developmental stages and therefore cannot establish the anatomical route or developmental mechanism of transovarial transmission in *H. longicornis*. In the transovarial lineage, some Eggs‐1 pools were PCR‐negative, whereas all tested Larvae‐1 pools were PCR‐positive. Because PCR was performed on different pooled subsets and the pool sizes differed between eggs and larvae, this pattern should be interpreted cautiously. Possible explanations include heterogeneous parasite distribution among egg batches, parasite burdens below the PCR detection threshold in some egg pools, or differences in detectability between the egg and larval stages. The pool‐level pattern does not, by itself, demonstrate parasite proliferation during embryonic development or larval hatching. Further studies using quantitative PCR and tissue localization approaches are needed to determine the parasite burden and developmental dynamics during oviposition, embryogenesis, and larval hatching.

*H. longicornis* feeds on a broad range of vertebrate hosts, including lagomorphs and livestock that are not considered susceptible to *B. gibsoni* [14, 16]. In the present laboratory experiments, ticks from the PCR‐positive transovarial F1 lineage transmitted *B. gibsoni* to splenectomized recipient dogs after one or two developmental stage feedings on rabbits. Thus, under the tested laboratory conditions, feeding on non‐susceptible rabbits did not eliminate transmission competence in the corresponding experimental lineages. The apparent reduction in pool‐level PCR positivity following rabbit feeding could reflect parasite loss from ticks, including potential release during blood feeding, or parasite burdens below the detection threshold; however, because different pooled subsets were examined and pool sizes differed among developmental stages, these possibilities cannot be distinguished from sampling effects, and the pool‐level values cannot be interpreted as changes in individual tick infection prevalence or parasite burden. Conversely, feeding on a susceptible host may provide conditions that favor parasite maintenance or amplification within ticks, although this hypothesis was not tested in the present study and requires quantitative validation. Whether rabbits or other non‐susceptible wild hosts act as ecological bridges in natural transmission cycles remains to be determined through field‐based investigation.

In contrast, no evidence of post‐molt transmission to splenectomized recipient dogs was observed after larval or nymphal acquisition feeding under the tested laboratory conditions. Donor dog 2 was smear‐positive at the time of tick placement and remained smear‐positive throughout the engorged tick collection period, indicating that the larvae and nymphs fed under parasitemic conditions. However, the engorged acquisition‐stage larvae and nymphs were not examined before molting. Accordingly, the present study could not determine the precise stage at which post‐molt transmission was restricted. One possible explanation is that the tick midgut and associated physiological or immune barriers, including the peritrophic matrix and midgut‐associated defenses, may influence parasite survival and dissemination after acquisition feeding [23]. The midgut‐resident symbiotic microbiota can modulate pathogen colonization in other tick–pathogen systems [24] and may therefore influence *B. gibsoni* survival, establishment, or dissemination, although this possibility was not examined in the present study. Developmental timing may also influence parasite establishment within the tick, but the relevant tissues and parasite stages were not examined in the present study. The smaller blood meals of larvae and nymphs may also result in a lower acquired parasite inoculum, which could reduce the probability of parasite survival or dissemination after molting. These possibilities remain hypotheses and require direct evaluation using quantitative PCR to compare parasite burdens before and after molting, together with tissue localization studies of the tick midgut, hemolymph, and salivary glands.

Comparisons with *B. ovis*, which can be transmitted transovarially by *Rhipicephalus bursa*, and *B. microti*, which are maintained through transstadial transmission by *Ixodes* spp., illustrate that transmission strategies differ among the *Babesia* species [25, 26]. Transovarial transmission could, in principle, reduce the need for repeated parasite acquisition from infected vertebrate hosts during the development of an infected tick lineage [13]. From a practical perspective, the ability of the tested transovarial F1 lineage to transmit at multiple developmental stages suggests that tick control and environmental management may complement host‐directed prevention measures [27]. Because transmission was observed from the larval, nymphal, and adult stages of the tested F1 lineage, questing ticks at multiple developmental stages may represent potential sources of infection under comparable conditions. Once a transovarially infected tick lineage has been established, dogs could remain exposed to potentially infectious ticks during periods when infected canine hosts are not locally detected.

Several limitations should be considered when interpreting these findings. The study was designed as a qualitative functional transmission experiment and was not intended to estimate individual tick infection prevalence or stage‐specific transmission probability. Splenectomized dogs provided a highly susceptible model for detecting transmission, but infection dynamics in this model may differ from those in immunocompetent dogs. One recipient dog was used for each tick stage or experimental lineage, limiting biological replication and preventing quantitative estimation of the transmission probability. Peak donor dog parasitemia was not experimentally synchronized with the rapid feeding phase of each individual tick. Nevertheless, the group‐level overall infestation periods, from tick placement to the final recovery of fully engorged ticks, encompassed the observed parasitemia peaks in both donor dogs, and both dogs remained smear‐positive throughout the relevant acquisition feeding periods. Thus, although acquisition feeding occurred under microscopically detectable parasitemia, the precise parasitemia encountered by each individual tick during its rapid feeding phase could not be determined. PCR screening was performed on pooled tick samples with different pool sizes, and nested PCR rather than quantitative PCR was used; therefore, individual tick infection prevalence, parasite burden, and quantitative differences among developmental stages could not be determined. Transmission competence was assessed only through the F1 larval, nymphal, and adult stages, and transmission from engorged F1 adult females to F2 eggs or larvae was not evaluated. All experiments used a single *Babesia*‐free parthenogenetic *H. longicornis* colony, whereas parthenogenetic and bisexual populations differ in ploidy, reproductive biology, genetic background, and pathogen‐associated traits [28, 29]. The findings should therefore be interpreted in the context of the *B. gibsoni* WH II strain, the tested parthenogenetic tick colony, controlled laboratory conditions, and the splenectomized recipient dog model. Replicated studies using quantitative PCR, tissue‐localization approaches, immunocompetent recipient dogs, and additional tick populations are needed to determine the broader biological relevance of these transmission patterns.

## 5. Conclusions

Under the tested laboratory conditions, this study provides evidence for the transovarial transmission of *B. gibsoni* by the parthenogenetic *H. longicornis* colony used in this study. Ticks from a PCR‐positive transovarial F1 lineage transmitted *B. gibsoni* to splenectomized recipient dogs at the larval, nymphal, and adult stages and retained transmission competence after one or two developmental‐stage feedings on non‐susceptible rabbits. In contrast, no evidence of post‐molt transmission to recipient dogs was observed after larval or nymphal acquisition feeding. These findings indicate that transovarially infected tick lineages may remain relevant transmission sources across multiple developmental stages and support the inclusion of tick control and environmental management alongside host‐directed prevention measures. The broader relevance of these transmission patterns should be evaluated in immunocompetent dogs and additional parthenogenetic and bisexual *H. longicornis* populations from geographically distinct origins.

## Author Contributions

**Yaxin Zheng:** methodology, data curation, investigation, writing – original draft. **Fangwei Chen**: data curation, investigation. **Jianyu Wang and Sen Wang**: investigation. **Zhen Han**: writing – review and editing. **Muxiao Li**: resources. **Fen Du and Junlong Zhao**: conceptualization, funding acquisition, project administration, resources, supervision. **Lan He**: conceptualization, funding acquisition, project administration, resources, supervision, validation, writing – review and editing.

## Funding

This work was supported by the National Key Research and Development Program of China (Grant 2024YFD1800100), the National Natural Science Foundation of China (Grant 32473058), the Fundamental Research Funds for the Central Universities in China (Grants 2662025DKPY013 and 2662025DKPY004), and the Tarim University—Huazhong Agricultural University Joint Fund (Grant HNLH202502).

## Ethics Statement

This study was approved by the Scientific Ethics Committee of Huazhong Agricultural University (Approval Number HZAUDO‐2025‐0022). The Beagle dogs utilized in this research were legally procured from licensed commercial breeders, strictly complying with all relevant ethical guidelines prior to the study. To ensure the smooth execution of surgical procedures and minimize animal distress, general anesthesia was induced and maintained using Zoletil. Postoperatively, all dogs were administered meloxicam for analgesia to effectively alleviate pain and reduce stress. All surgical interventions were performed by a professional veterinary team in strict adherence to established animal welfare and ethical standards. Comprehensive postoperative care was provided, which included continuous monitoring of the animals’ recovery, provision of a quiet environment with appropriate nutrition, and prophylactic measures against postoperative infections, thereby ensuring timely and optimal care.

## Conflicts of Interest

The authors declare no conflicts of interest.

## Supporting Information

Additional supporting information can be found online in the Supporting Information section.

## Supporting information


**Supporting Information 1** Additional file 2: Table S1: Detailed information on donor‐dog parasitemia and tick acquisition, pool‐level PCR screening of experimental tick lineages, and transmission outcomes in recipient dogs.

**Supporting Information 2** Additional file 1: Figures S1 and S2: PCR detection of *B. gibsoni* in ticks and dogs across experimental transmission lines.

## Data Availability

The data supporting the findings of this study are available in the Supporting Information of this article.
