# Supplementary material for: Laboratory Evidence for Transovarial Transmission of Babesia gibsoni by Haemaphysalis longicornis
Source: Transbound Emerg Dis. 2026 Sep 15;2026:5585506. doi: 10.1155/tbed/5585506 (PMC13578936; doi:10.1155/tbed/5585506)
Supplement: Supplementary file 1 [file TBED-2026-5585506-s002.pdf]

Additional file 1: Figures S1–S2. PCR detection of *Babesia gibsoni* in ticks and dogs across experimental transmission lines.

Figure S1. PCR detection of *B. gibsoni* in transovarial F1 tick lineages and corresponding recipient dogs.

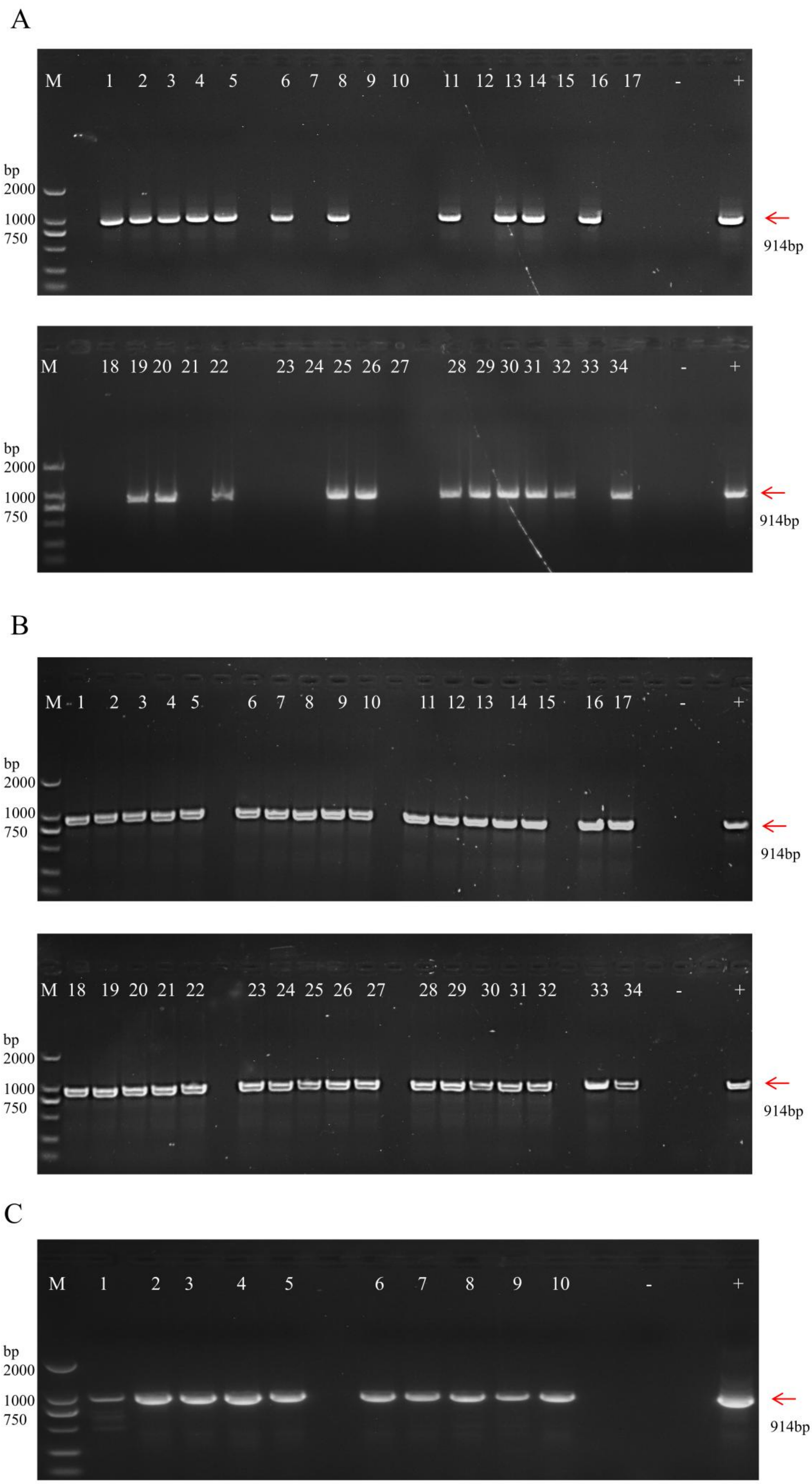
A
M 1 2 3 4 5 6 7 8 9 10 11 12 13 14 15 16 17 - +
bp
2000
1000
750
914bp
M 18 19 20 21 22 23 24 25 26 27 28 29 30 31 32 33 34 - +
bp
2000
1000
750
914bp
B
M 1 2 3 4 5 6 7 8 9 10 11 12 13 14 15 16 17 - +
bp
2000
1000
750
914bp
M 18 19 20 21 22 23 24 25 26 27 28 29 30 31 32 33 34 - +
bp
2000
1000
750
914bp
C
M 1 2 3 4 5 6 7 8 9 10 - +
bp
2000
1000
750
914bp

D

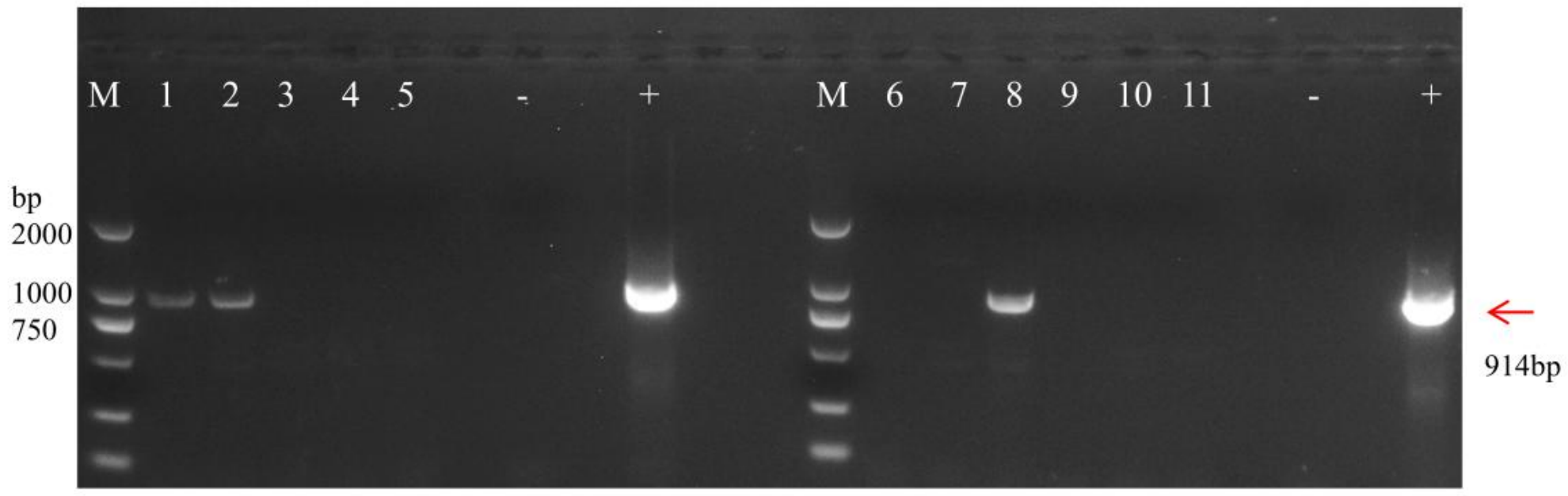
M 1 2 3 4 5 - +
M 6 7 8 9 10 11 - +
bp
2000
1000
750
914bp

E

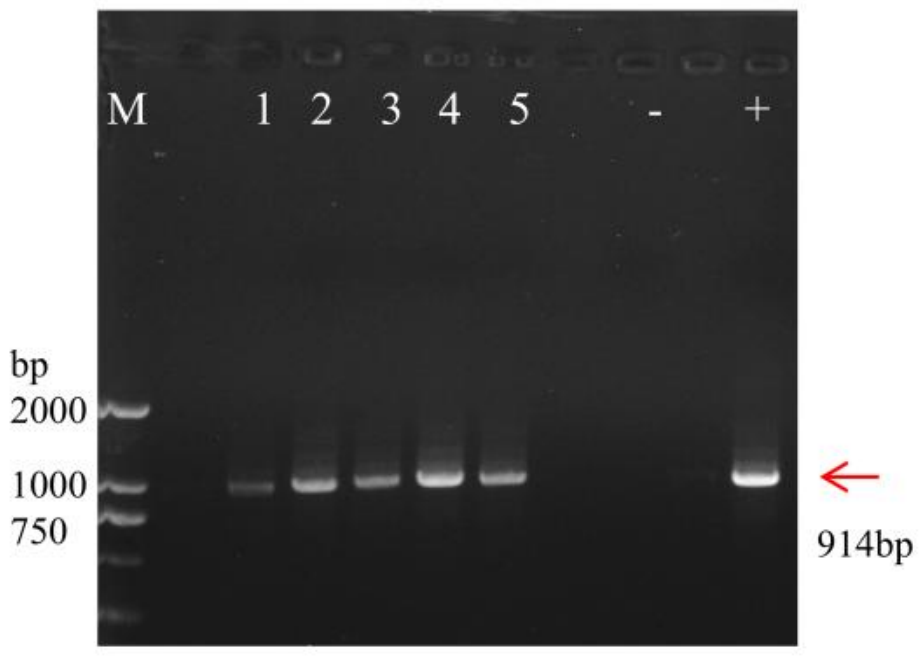
M 1 2 3 4 5 - +
bp
2000
1000
750
914bp

F

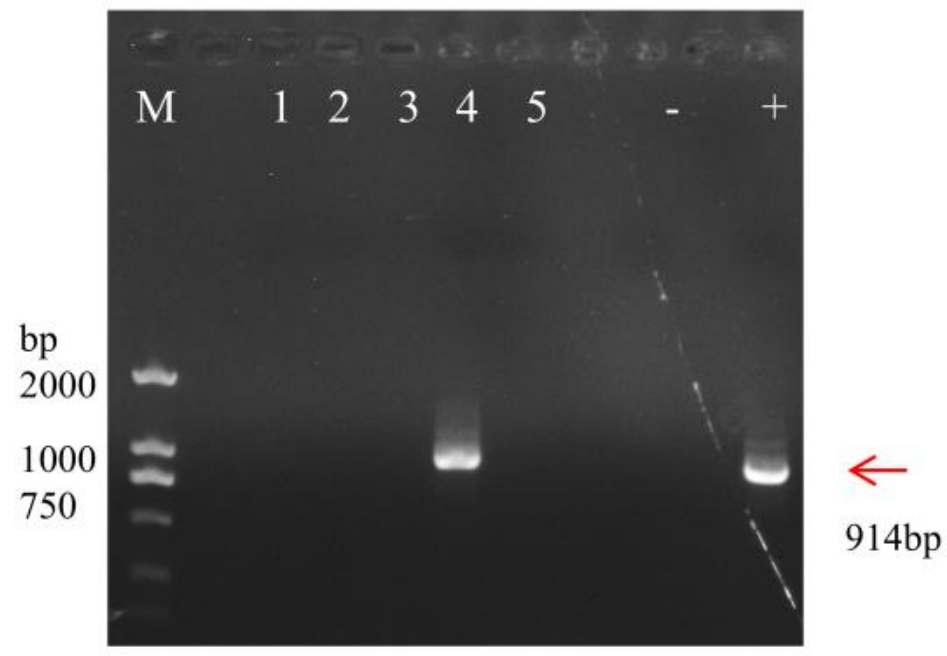
M 1 2 3 4 5 - +
bp
2000
1000
750
914bp

G

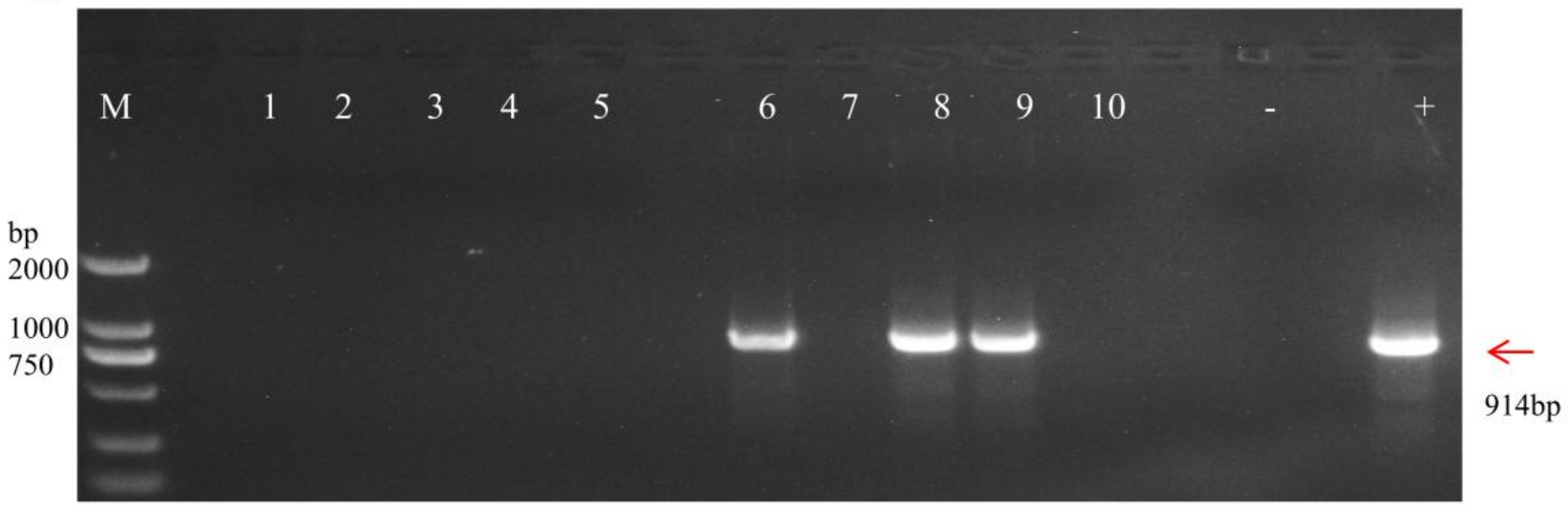
M 1 2 3 4 5 6 7 8 9 10 - +
bp
2000
1000
750
914bp

H

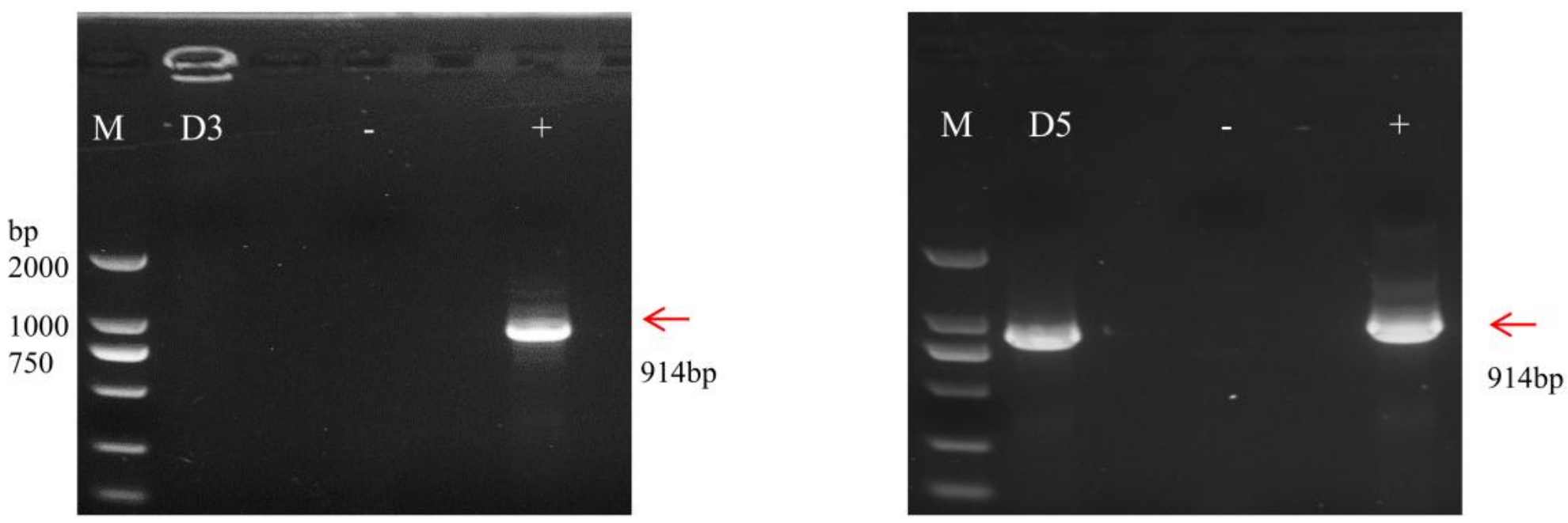
M D3 - +
bp
2000
1000
750
914bp
M D5 - +
914bp

I

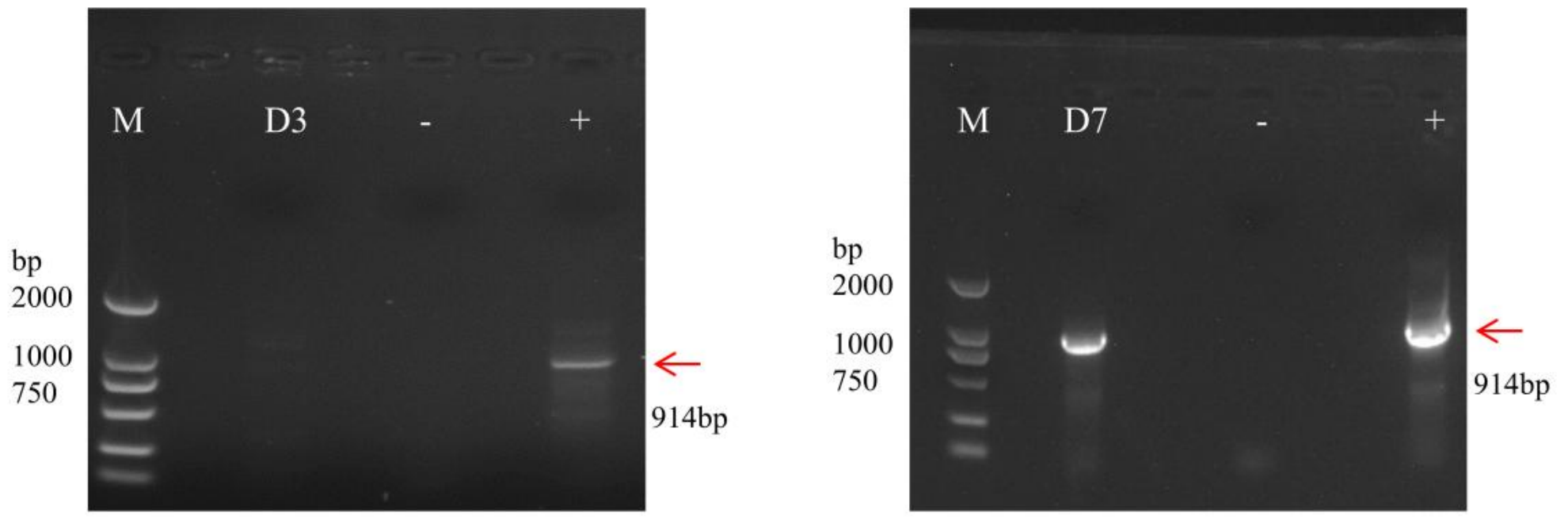
M
D3
-
+
bp
2000
1000
750
914bp
M
D7
-
+
bp
2000
1000
750
914bp

J

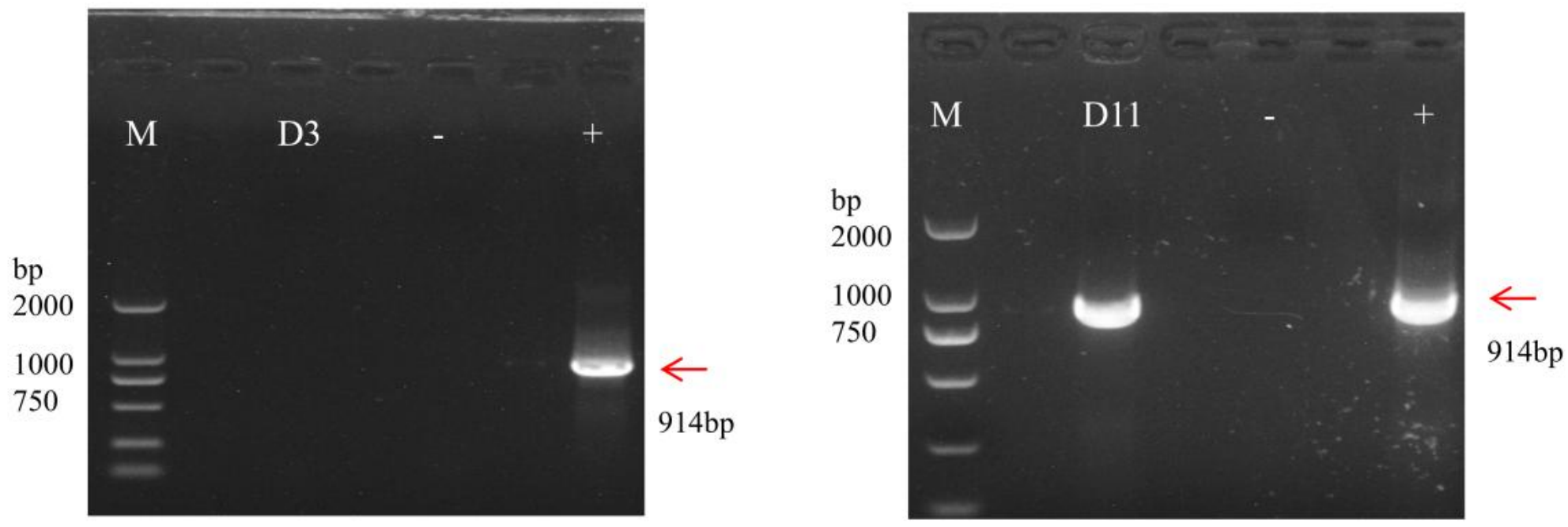
M
D3
-
+
bp
2000
1000
750
914bp
M
D11
-
+
bp
2000
1000
750
914bp

K

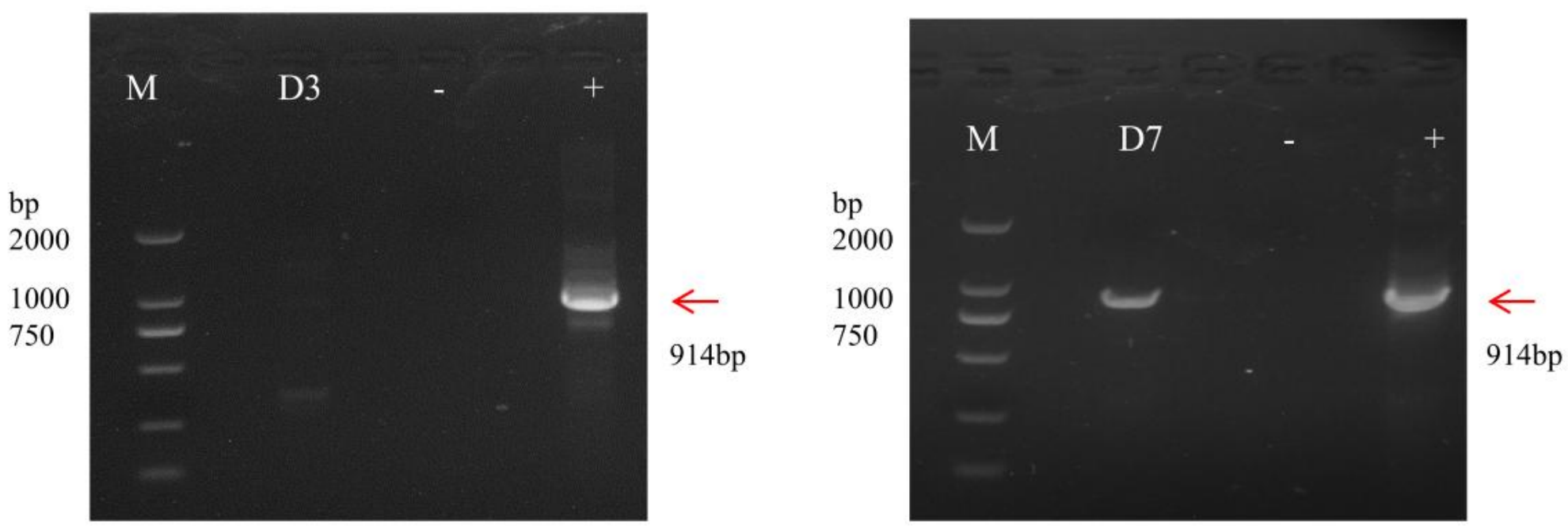
M
D3
-
+
bp
2000
1000
750
914bp
M
D7
-
+
bp
2000
1000
750
914bp

L

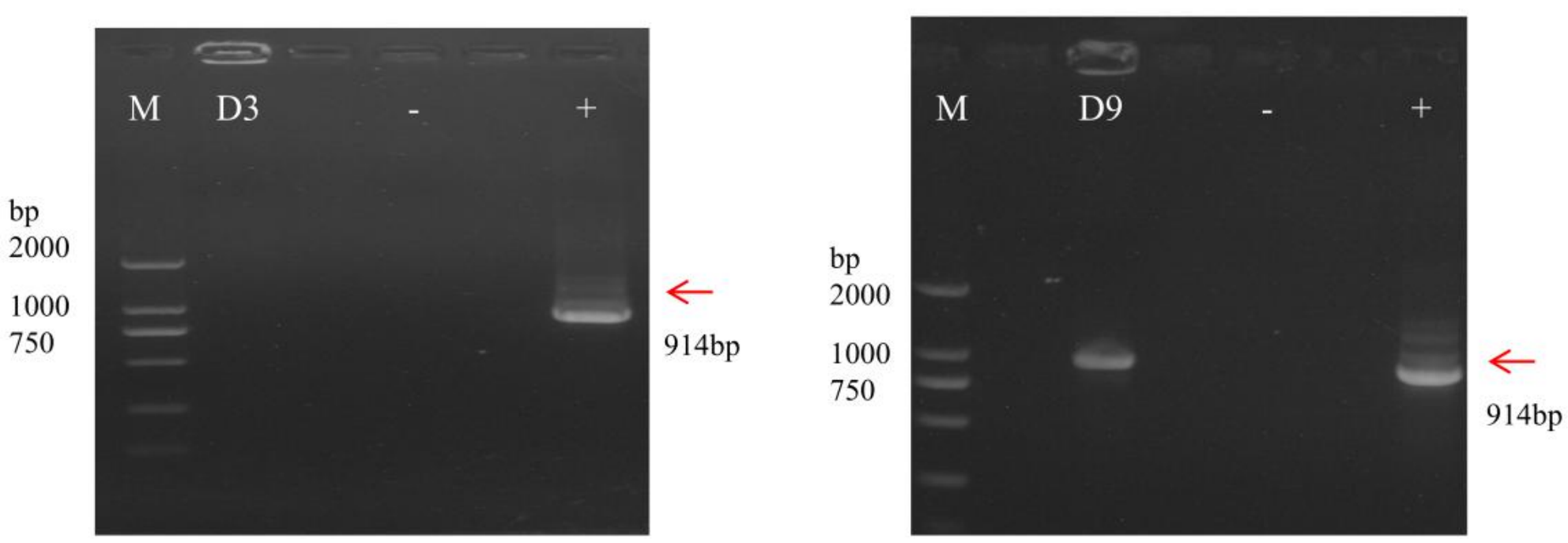
M
D3
-
+
bp
2000
1000
750
914bp
M
D9
-
+
bp
2000
1000
750
914bp

(A–D) Pooled PCR detection of *B. gibsoni* in the transovarial F1 lineage derived from adult females that fed on an infected donor dog: (A) Eggs-1 after oviposition, 22/34 PCR-positive pools; (B) unfed Larvae-1 after hatching, 34/34 PCR-positive pools; (C) unfed post-molt Nymphs-1, 10/10 PCR-positive pools; and (D) unfed post-molt Adults-1, 3/11 PCR-positive pools. (E–G) Pooled PCR detection in the transovarial F1 lineage used for rabbit passage: (E) unfed Larvae-2 before rabbit feeding, 5/5 PCR-positive pools; (F) unfed post-molt Nymphs-2 derived from larvae that had fed on rabbits, 1/5 PCR-positive pools; and (G) unfed post-molt Adults-2 derived from nymphs that had fed on rabbits, 3/10 PCR-positive pools. (H–L) First PCR-positive blood samples from recipient dogs infested with ticks from the corresponding experimental lineages: (H) T01, infested with Larvae-1, 5 dpi; (I) T02, infested with Nymphs-1, 7 dpi; (J) T03, infested with Adults-1, 11 dpi; (K) T04, infested with Nymphs-2, 7 dpi; and (L) T05, infested with Adults-2, 9 dpi. The expected amplicon size was 914 bp. M, DNA marker; (+), positive PCR control; (−), batch-matched mock negative extraction control. Numbered lanes in panels A–G indicate individual pooled tick samples. Values for tick samples are presented as the number of PCR-positive pools/total pools tested. dpi, days post-infestation.

Figure S2. PCR detection of *B. gibsoni* in ticks and dogs during post-molt transmission assessment following larval or nymphal acquisition feeding.

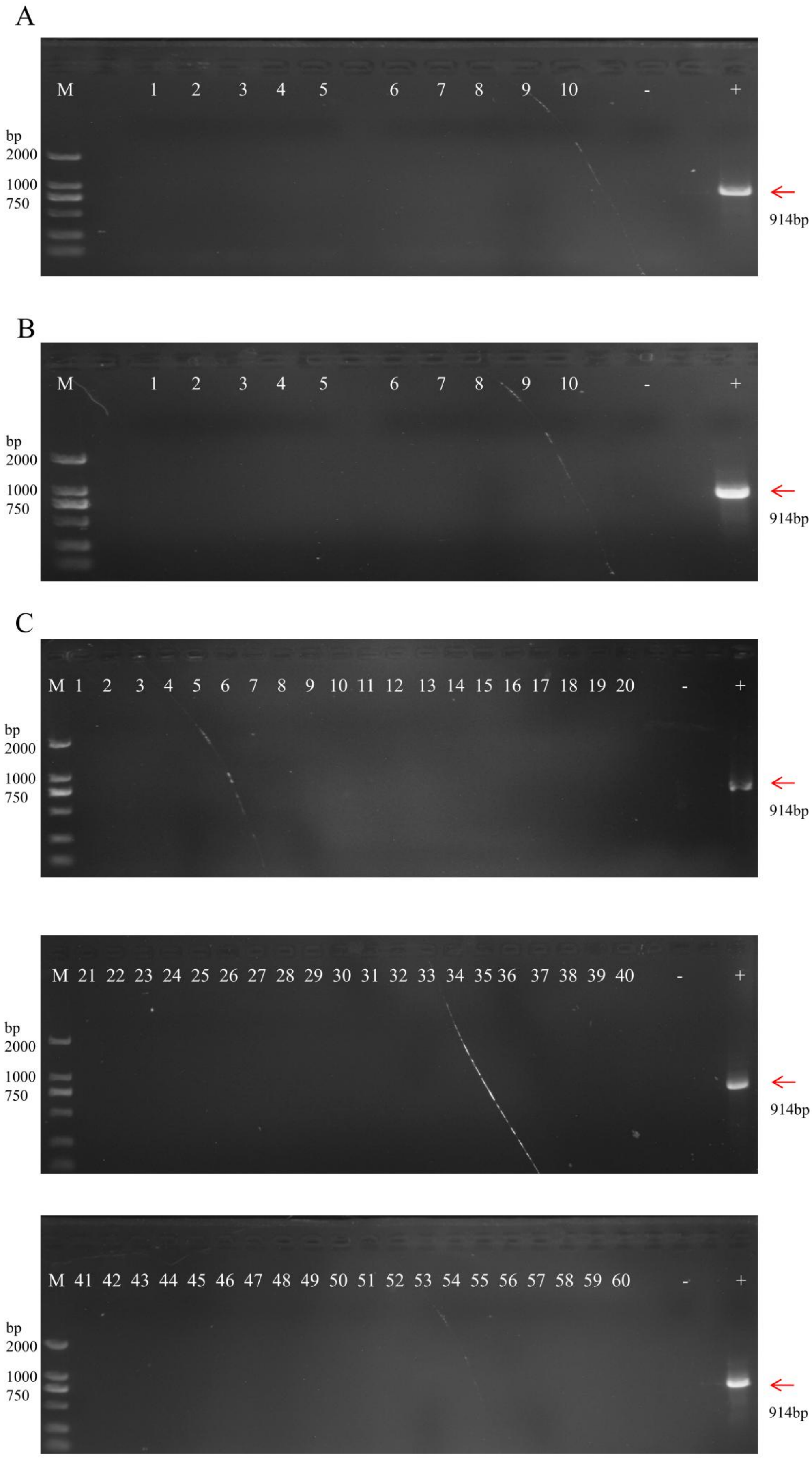

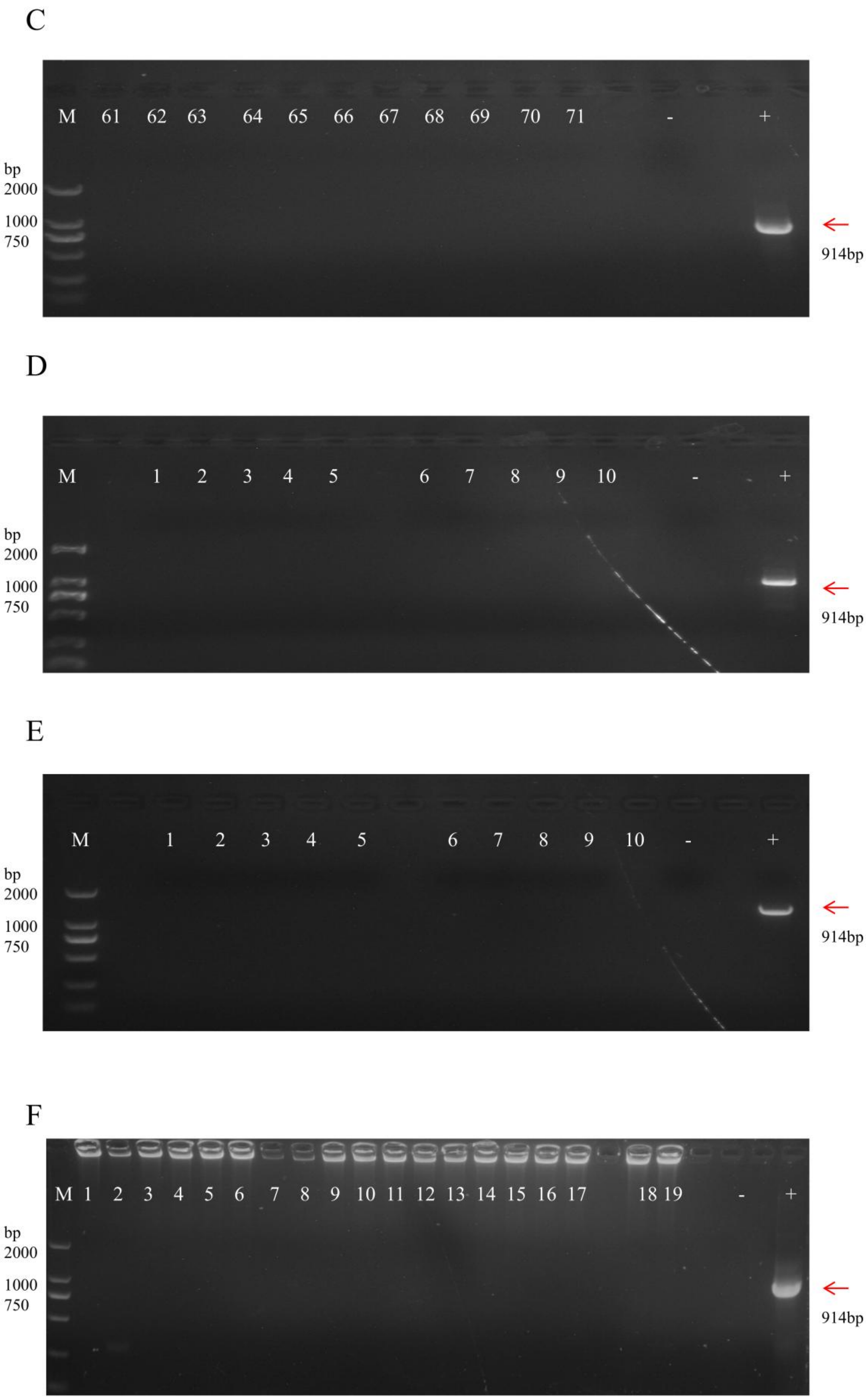
C
M 61 62 63 64 65 66 67 68 69 70 71 - +
bp
2000
1000
750
914bp
D
M 1 2 3 4 5 6 7 8 9 10 - +
bp
2000
1000
750
914bp
E
M 1 2 3 4 5 6 7 8 9 10 - +
bp
2000
1000
750
914bp
F
M 1 2 3 4 5 6 7 8 9 10 11 12 13 14 15 16 17 18 19 - +
bp
2000
1000
750
914bp

F

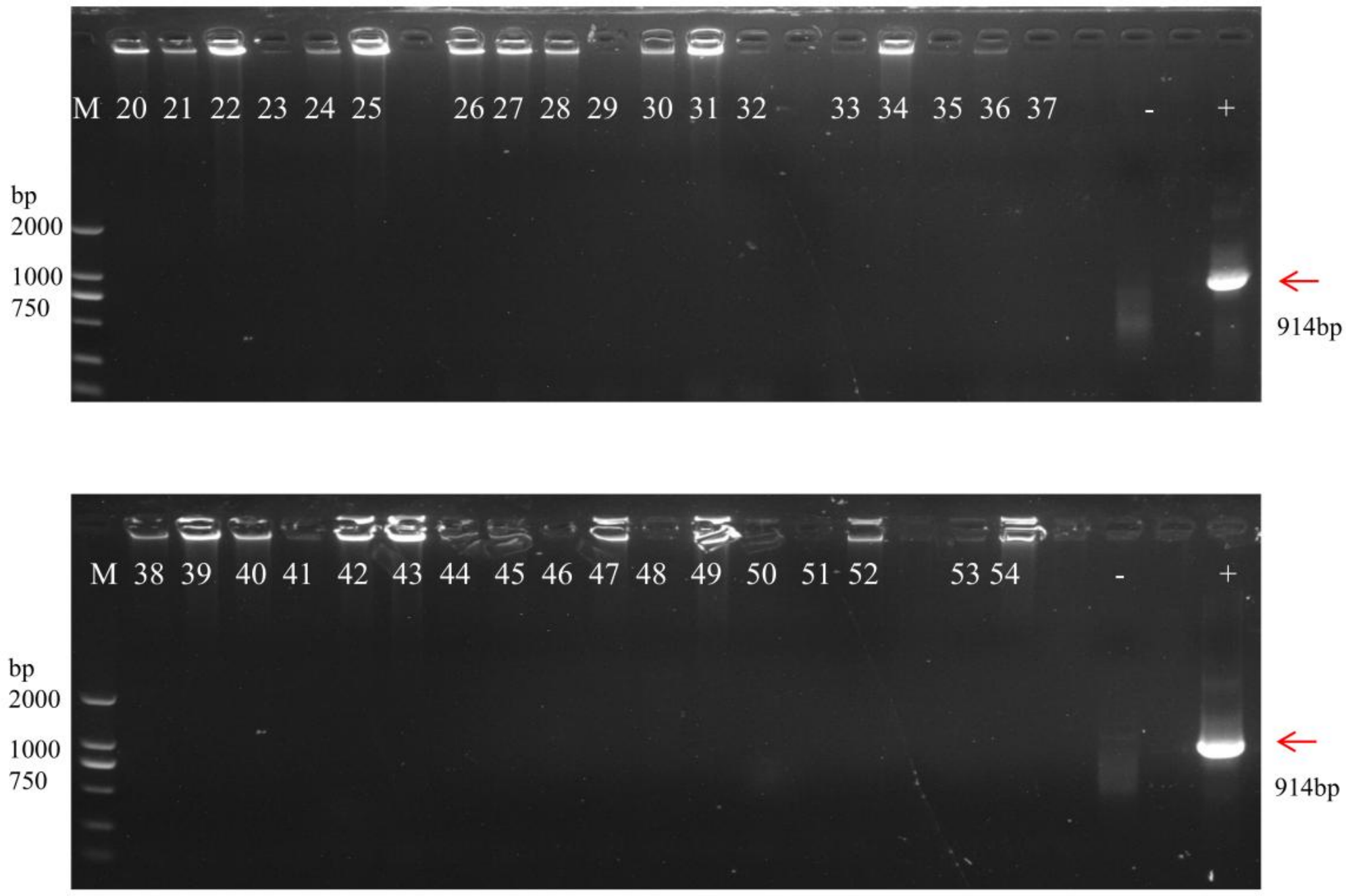
M 20 21 22 23 24 25 26 27 28 29 30 31 32 33 34 35 36 37 - +
bp
2000
1000
750
914bp
M 38 39 40 41 42 43 44 45 46 47 48 49 50 51 52 53 54 - +
bp
2000
1000
750
914bp

G

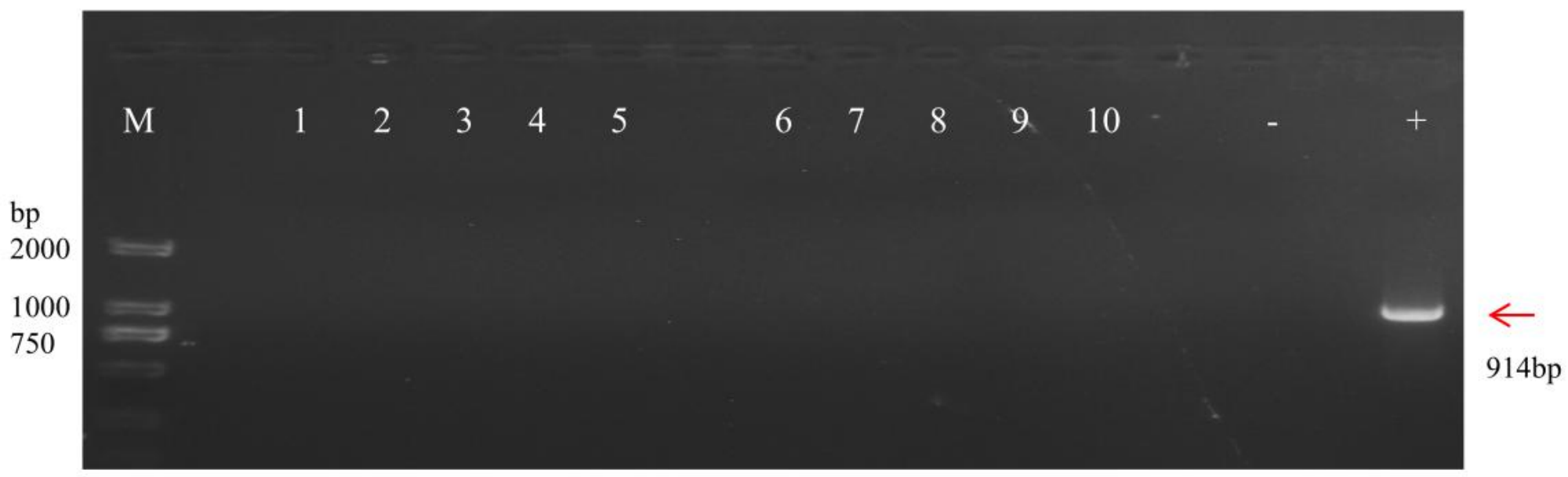
M 1 2 3 4 5 6 7 8 9 10 - +
bp
2000
1000
750
914bp

H

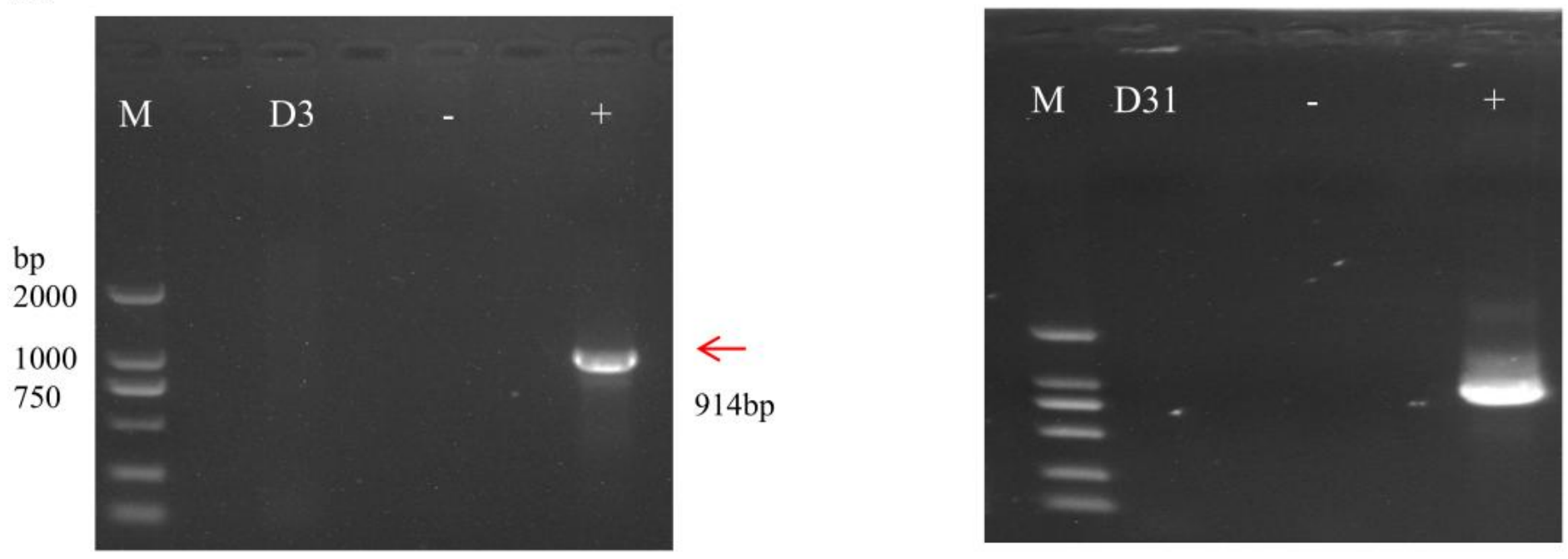
M D3 - +
bp
2000
1000
750
914bp
M D31 - +

I

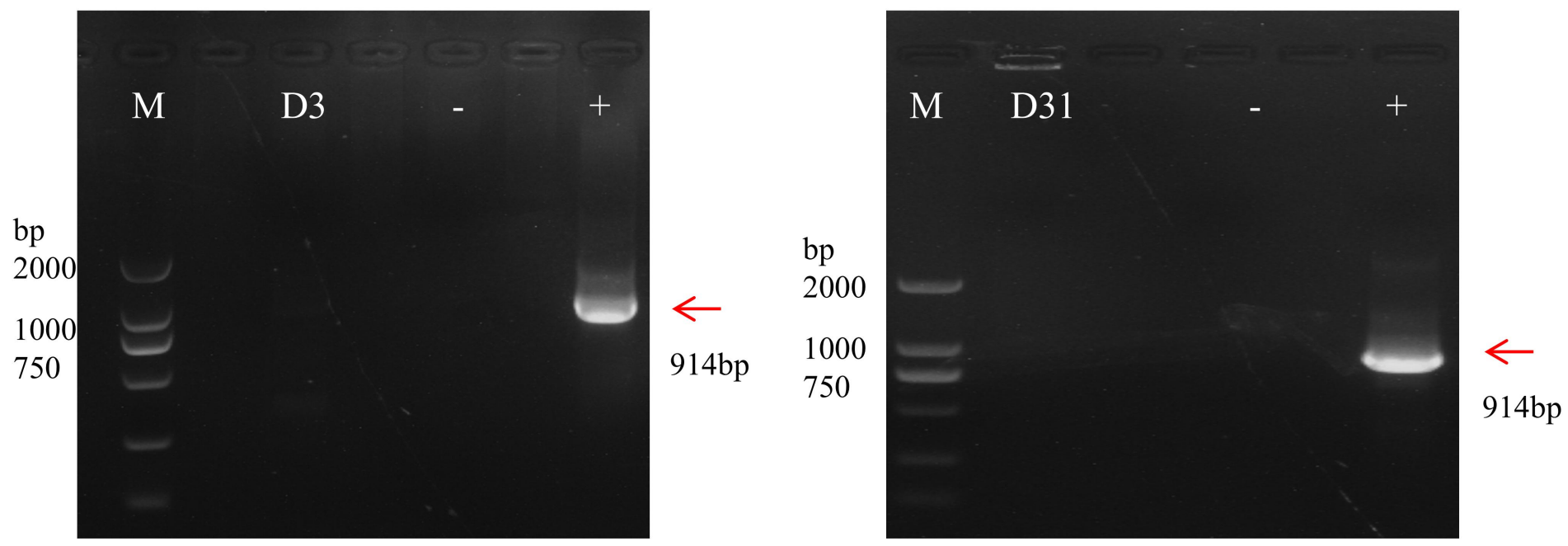
M
D3
-
+
bp
2000
1000
750
914bp
M
D31
-
+
bp
2000
1000
750
914bp

J

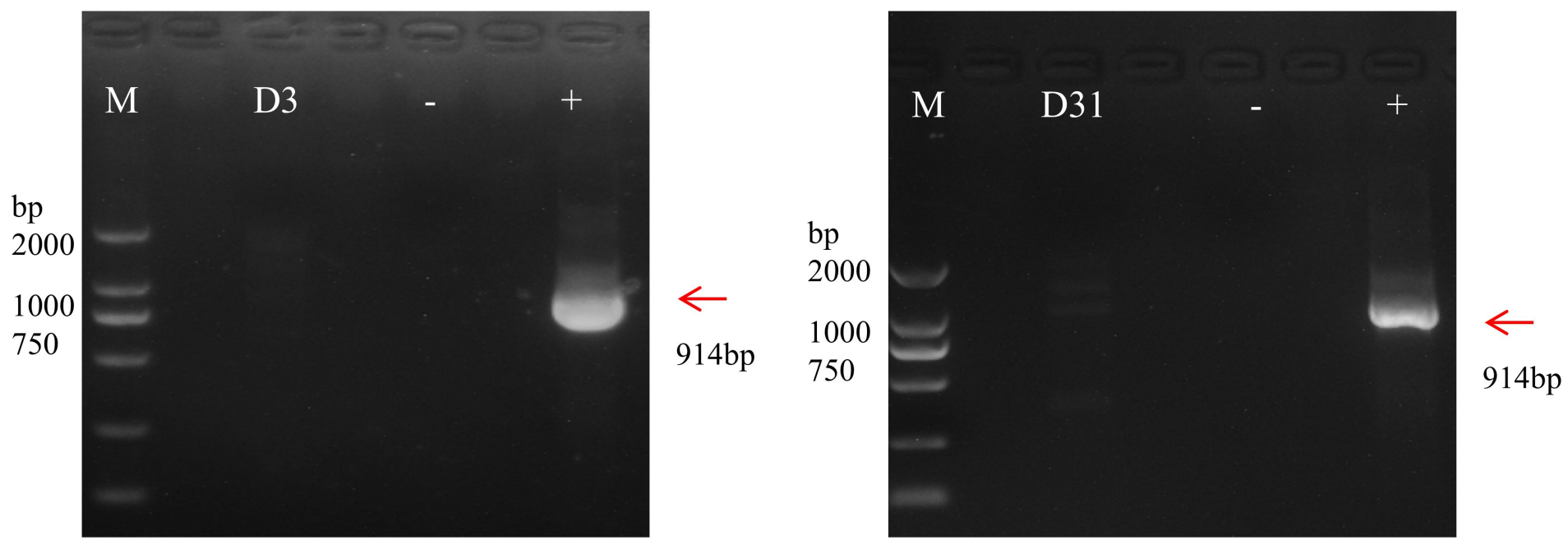
M
D3
-
+
bp
2000
1000
750
914bp
M
D31
-
+
bp
2000
1000
750
914bp

K

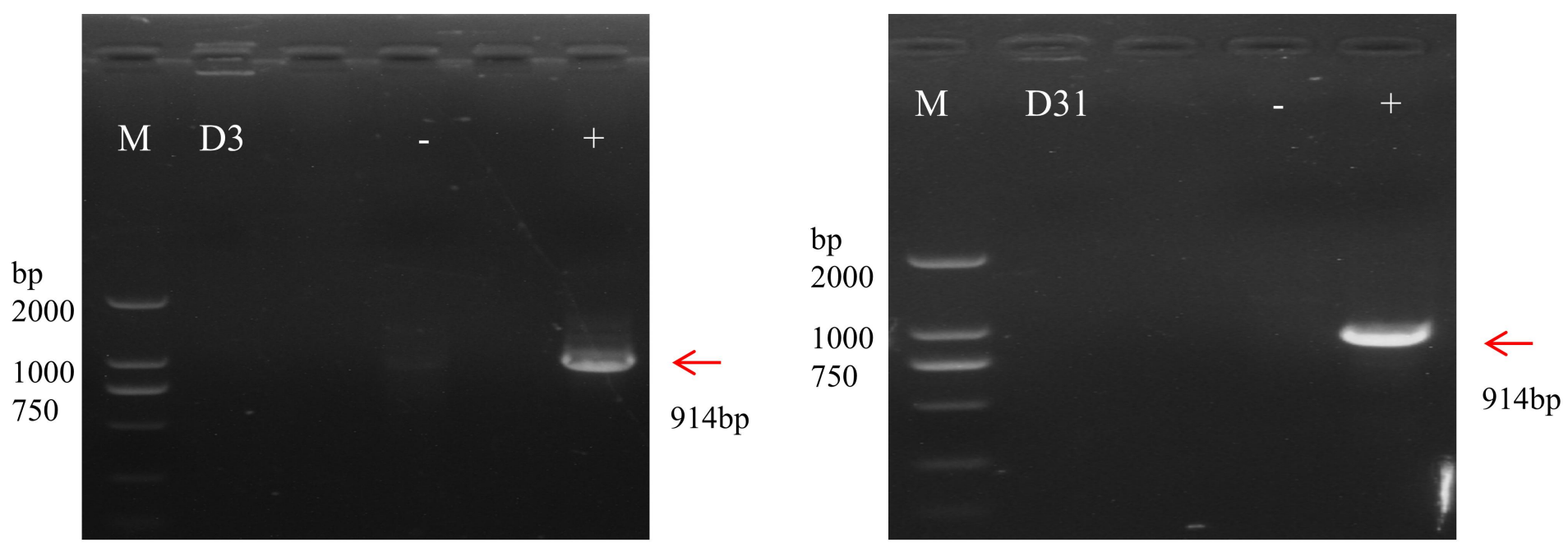
M
D3
-
+
bp
2000
1000
750
914bp
M
D31
-
+
bp
2000
1000
750
914bp

L

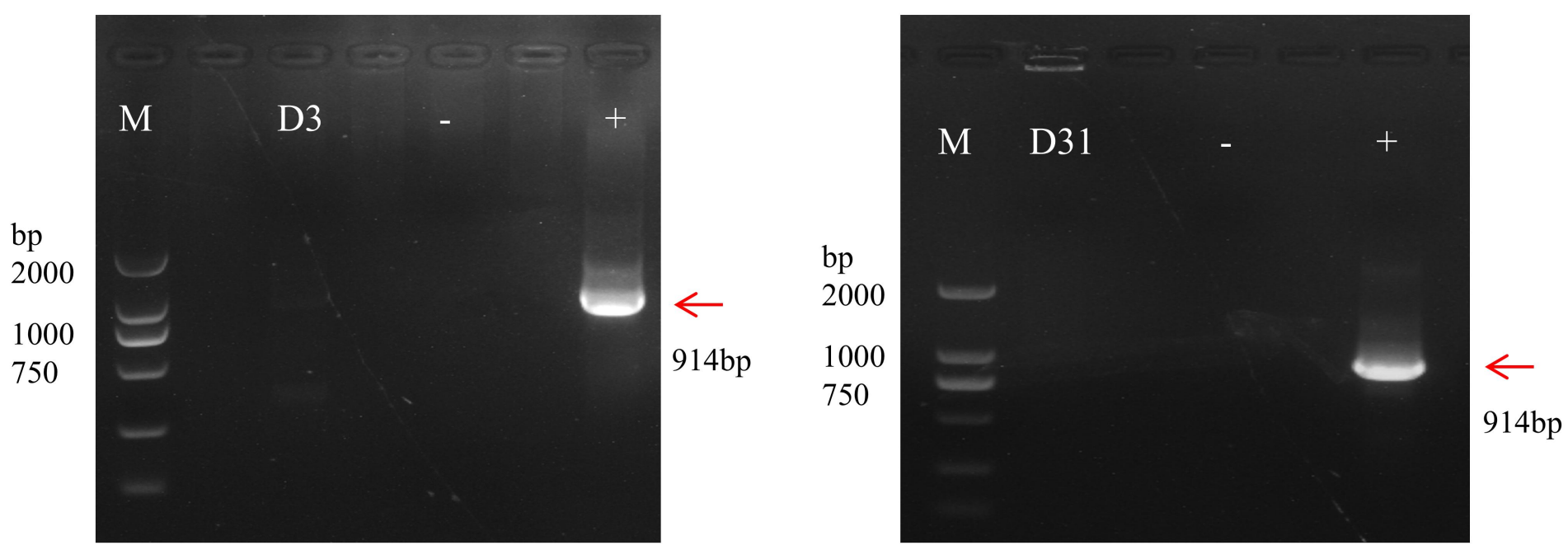
M
D3
-
+
bp
2000
1000
750
914bp
M
D31
-
+
bp
2000
1000
750
914bp

(A–D) Pooled PCR screening of the larval-acquisition line: (A) unfed post-molt Nymphs-3 derived from Babesia-free larvae that had fed on an infected donor dog, 0/10 PCR-positive pools; (B) unfed post-molt Adults-3 derived from Nymphs-3 recovered from recipient dog T06, 0/10 PCR-positive pools; (C) Eggs-2 collected after oviposition by engorged Adults-3 recovered from recipient dog T07, 0/71 PCR-positive pools; and (D) unfed Larvae-3 after hatching from Eggs-2, 0/10 PCR-positive pools. (E–G) Pooled PCR screening of the nymphal-acquisition line: (E) unfed post-molt Adults-4 derived from Babesia-free nymphs that had fed on an infected donor dog, 0/10 PCR-positive pools; (F) Eggs-3 collected after oviposition by engorged Adults-4 recovered from recipient dog T09, 0/54 PCR-positive pools; and (G) unfed Larvae-4 after hatching from Eggs-3, 0/10 PCR-positive pools. (H–L) PCR monitoring of recipient dogs infested with ticks from the corresponding experimental lineages: (H) T06, infested with Nymphs-3; (I) T07, infested with Adults-3; (J) T08, infested with Larvae-3; (K) T09, infested with Adults-4; and (L) T10, infested with Larvae-4. All recipient dogs remained PCR-negative through 31 days post-infestation. The expected amplicon size was 914 bp. M, DNA marker; (+), positive PCR control; (−), batch-matched mock negative extraction control. Numbered lanes in panels A–G indicate individual pooled tick samples. Values for tick samples are presented as the number of PCR-positive pools/total pools tested.
